# Single Component Dye-Sensitized Solar Cells Enabled by Copper Chemistry: Introduction of the Retro Cell

**DOI:** 10.1021/acs.energyfuels.4c06413

**Published:** 2025-03-07

**Authors:** Samhita Kaushik, Michael A. Adesanya, Thomas W. Hamann

**Affiliations:** Department of Chemistry, Michigan State University, East Lansing, Michigan 48824-1322, United States

## Abstract

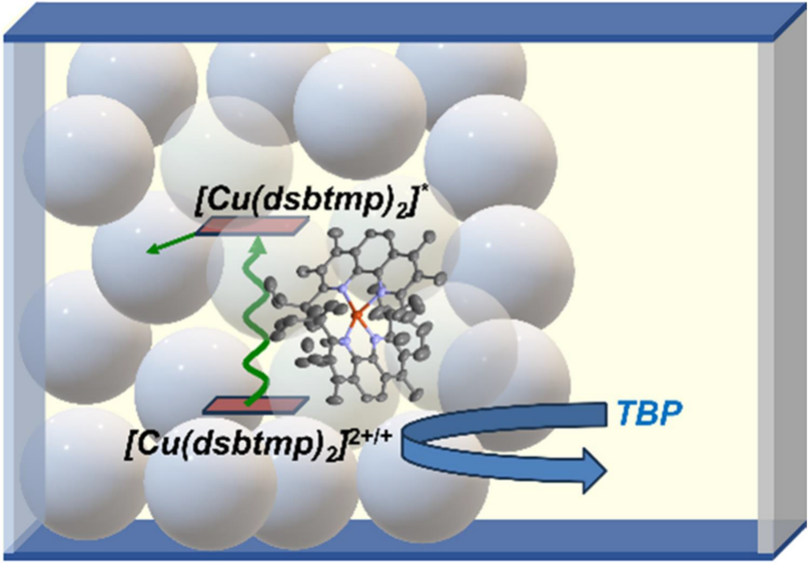

The possibility of utilizing a single molecule to act as both a chromophore and a redox shuttle in a new configuration of a dye-sensitized solar cell (DSSC) is investigated. This design, termed a retro cell, exploits the copper chromophore, [Cu(dsbtmp)_2_]^+^ (dsbtmp = bis(2,9-di(*sec*-butyl)-3,4,7,8-tetramethyl-1,10-phenanthroline), which has been shown to have excited state lifetimes in excess of a microsecond, enabling sensitization of TiO_2_ while dissolved in solution. The oxidized chromophore can then diffuse to the counter electrode to be regenerated. This concept simplifies the device components and fabrication and eliminates a charge transfer step compared to that of traditional DSSCs. Initial investigations show the concept is viable; however, the performance is suboptimal. We have found the addition of 4-*tert*-butylpyridine (TBP) to the electrolyte plays a crucial role in enabling solar energy conversion. Evidence of TBP displacing one of the dsbtmp ligands in the oxidized [Cu(dsbtmp)_2_]^2+^ complex has been presented, which likely plays an important role in reducing recombination and enabling charge collection. The performance-limiting steps and routes to improved performance and viability of a retro cell are further discussed.

## Introduction

Dye-sensitized solar cells (DSSCs) are an attractive modular system capable of harnessing solar energy and indoor light for electricity generation, as well as serving as the platform for photoelectrosynthsis cells to store solar energy in chemical bonds.^1−3^ Since O’Regan and Graetzel’s seminal report using nanocrystalline TiO_2_ electrodes to enable good light absorption and power conversion efficiencies (PCEs), there has been a concerted effort to optimize each component in the cell to further advance DSSCs.^3−6^ Ruthenium-based dyes such as N719,^4,7^ N3,^8^ and black dye^9^ are some of the best-known photosensitizers in DSSCs, which gave excellent PCE with the iodide/triiodide redox couple but are not as compatible with transition metal-based redox couples. The scarcity and cost of ruthenium motivated the exploration of Earth-abundant alternatives. Sauvage and colleagues reported the utilization of copper complexes as sensitizers, driven by the similarity in photophysical properties between Cu(I) and Ru(II) complexes.^10,11^ Recent years have seen a surge in research dedicated to leveraging inexpensive and environmentally friendly Cu(I) complexes as sensitizers in DSSCs.^12−21^ Odobel et. al synthesized a Cu(I) complex with 6,6′-dimesityl-2,2′-bipyridine-4,4′-dicarboxylic acid as the anchoring ligand and 4,4′-bis(N,N-diethylaminestyryl)-6,6′-dimethyl-2,2′-bipyridine as the ancillary ligand that resulted in a reported PCE of 4.66%.^22^ This PCE may be an overestimate as the active area was not masked,^23^ but nevertheless this report is a notable demonstration of first-row transition metal coordination complexes as sensitizers.^22^

In 2005, Fukuzumi and colleagues pioneered the use of copper complexes as redox shuttles in DSSCs. They evaluated the photovoltaic properties of DSCCs employing the N719 dye with a range of complexes, including [Cu(phen)_2_]^2+/+^ and [Cu(dmp)_2_]^2+/+^, where phen is phenanthroline and dmp is 2,9-dimethyl phenanthroline.^24^ Since then, significant advancements in the performance of Cu(II/I) redox mediators have been achieved through ligand structure tailoring, cosensitization strategies, and innovative device architectures.^25,26^ Notably, Grätzel et al. reported DSSCs utilizing a Cu(II)/Cu(I) redox-based electrolyte, achieving efficiencies of up to 15%.^27^ The use of Cu(II)/Cu(I) redox mediators has also allowed high indoor light conversion efficiencies, up to 40%, to be achieved.^28,29^ These redox shuttles are interesting because they can act as fast reducing agents, thus minimizing the energy penalty of the dye regeneration step; however, they are also slow enough acceptors to allow quantitative charge collection. We have previously shown that some Cu(II) complexes, including [Cu(dmbpy)_2_]^2+^ (dmbpy = 6,6′-dimethyl-2,2′-bipyridine), undergo a ligand exchange reaction with an exogenous base, e.g., 4-*tert*-butylpyridine (TBP), to form the slow electron acceptor [Cu(TBP)_4–6_]^2+^.^30^ The electrolyte thus contains the redox couple Cu(dmbpy)_2_]^+^/[Cu(TBP)_4–6_]^2+^; the combination of a fast electron donor and a slow electron acceptor explains some of the excellent overall performance of this redox system. In other studies, however, it was reported that TBP binds to the Cu(II) center but does not displace the bidentate phen ligand, and the ligand substitution reaction remains an active area of inquiry.^31^ We note that the Cu(I) redox shuttles can have significant competitive absorption due to metal-to-ligand charge transfer (MLCT) bands in the visible region, which is deleterious when employed as a redox mediator. Indeed, this strong absorbance is sometimes taken advantage of to utilize copper complexes as earth-abundant transition metal chromophores for DSSCs as noted above.^32^ With the goals of using earth-abundant metals for designing DSSCs and the knowledge of copper complexes being successful candidates as both photosensitizers and redox shuttles, the development of full-copper DSSCs was explored by several groups.^33,34^ Competitive absorption between the dye and the redox shuttle poses an intrinsic limitation on such systems, potentially hindering them from reaching their maximum efficiency.

**Figure 1:**
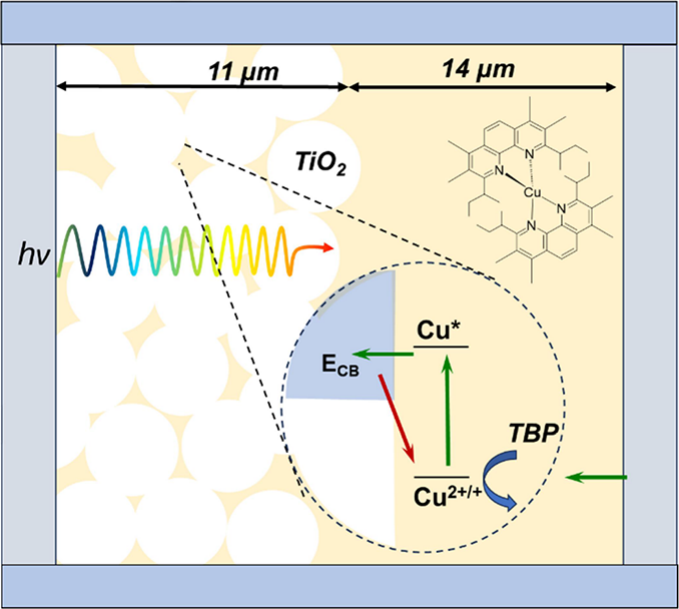
Schematic of a retro cell. Following light absorption by the copper complex in the mesoporous photoanode, the excited chromophore (E* = −1.46 V vs Fc^+/0^) will diffuse to the TiO_2_ surface and inject an electron into the conduction band (*E*_CB_ = −1.161 to −1.336 V vs Fc in acetonitrile^41^), and the oxidized chromophore will diffuse to the counter electrode or be intercepted by TBP in solution.

In this work, we introduce an alternative concept, depicted in Figure 1, where we use a single copper complex dissolved in solution to act as both photosensitizer and redox shuttle. This is inspired by pioneering work by Clark and Sutin who investigated the sensitization of single crystal TiO_2_ electrodes using ruthenium tris(4,7-dimethyl-1,10-phenanthroline), [Ru(dmphen)_3_]^2+^ in solution.^35^ They found that photoexcited [Ru(dmphen)_3_]^2+^ within an effective diffusion distance of 59 nm, determined by the 1.74 μs excited state lifetime, τ, of the Ru sensitizer, injects electrons into the TiO_2_ conduction band (CB) with essentially unity quantum yield.^36^ The photocurrent was ultimately limited by the small fraction of excited sensitizer molecules within this diffusion distance of the flat electrode surface. Because modern DSSCs utilize a mesoporous TiO_2_ photoanode where the pore size is on the order of 10–20 nm, determined by the TiO_2_ nanoparticle size and preparation conditions, all photoexcited sensitizers with a μs lifetime would diffuse to a TiO_2_ surface and allow charge separation.^4^ Ruthenium polypyridyl complexes are good electron acceptors, however, and fast recombination limits charge collection for such systems.^37^ While band-bending in single-crystal TiO_2_ can inhibit such recombination losses, there is no band-bending in nanoparticle TiO_2_ electrodes, and recombination by interfacial electron transfer is a critical limitation. This is the reason that kinetically slow redox shuttles are generally utilized to reduce the oxidized chromophores and allow efficient charge collection.^38^

Castellano has reported a series of copper complexes with long excited state lifetimes and strong absorbance in the visible region analogous to ruthenium polypyridyl analogs. For example, [Cu(dsbtmp)_2_]^+^ (dsbtmp = bis(2,9-di(*sec*-butyl)-3,4,7,8-tetramethyl-1,10-phenanthroline) was shown to have excited-state lifetimes >1 μs in a variety of solvents.^39^ A molar absorptivity of 7400 M^–1^ cm^–1^ at 445 nm allows good visible light absorption within a ca. 10 μm TiO_2_ mesoporous electrode.^40^ These attributes suggest the possibility of using [Cu(dsbtmp)_2_]^+^ dissolved in a solution as a sensitizer for TiO_2_. Diffusion of oxidized [Cu(dsbtmp)_2_]^2+^ to the counter electrode would complete the circuit. Such a solar cell combines an old concept of charge separation from dissolved chromophores in contact with a modern configuration of a semiconductor photoanode, which we thus term a retro cell. The retro cell design is advantageous as it eliminates the energy loss of the dye regeneration step, eliminates the time-intensive dye absorption step, and may decrease instability due to dye desorption. In order to be successful, recombination losses to [Cu(dsbtmp)_2_]^2+^ must be minimized to allow for good charge collection. Herein, we report the first example of a retro cell and identify rate-limiting steps in photocurrent generation and future directions to advance the efficiency and application scope.

## Results and Discussion

**Figure 2:**
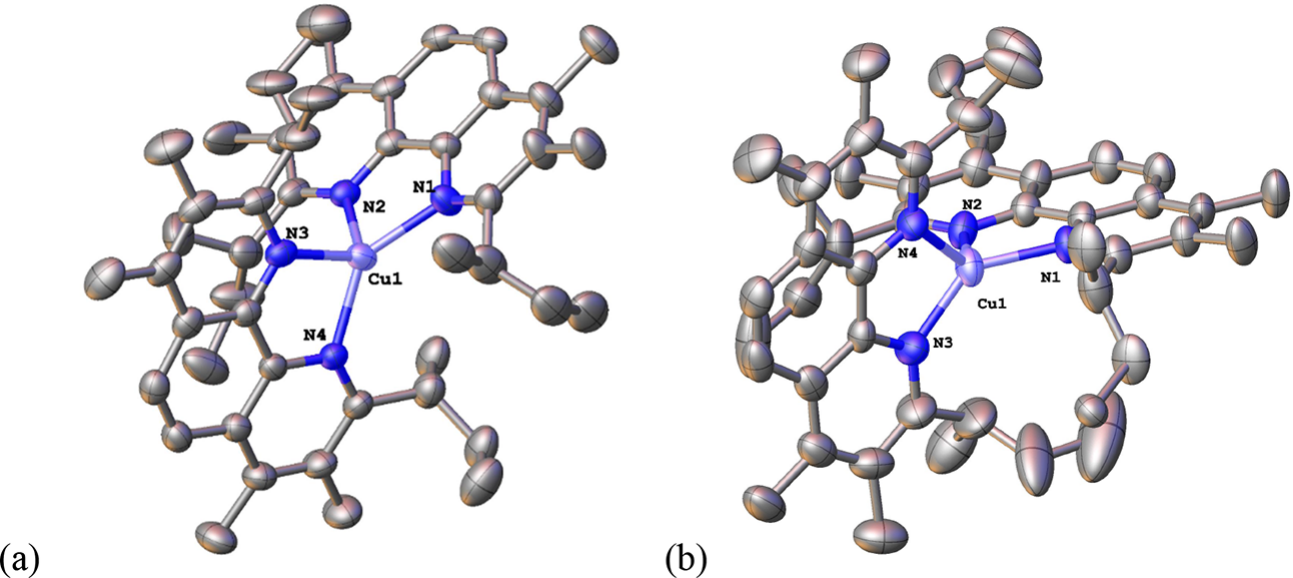
Single crystal X-ray structures with thermal ellipsoids drawn at 50% probability of (a) [Cu(dsbtmp)_2_](PF_6_) and (b) [Cu(dsbtmp)_2_](PF_6_)_2_. Hydrogen atoms and counterions are omitted for clarity.

The [Cu(dsbtmp)_2_]^+^ complex was synthesized according to a previously reported method.^39^ The Cu(II) form was prepared by oxidation with an equimolar solution of nitrosonium hexafluorophosphate, NOPF_6_, and [Cu(dsbtmp)_2_]^+^ in dichloromethane in an inert atmosphere. Synthetic methods and characterization are provided in the experimental section below, with additional details in the Supporting Information. The crystal structures of [Cu(dsbtmp)_2_]^+^ and [Cu(dsbtmp)_2_]^2+^ are shown below in Figure 2, with select bond lengths and angles provided in Table 1 below. Additional structures showing disorder in the *sec*-butyl groups and with hydrogen atoms included are shown in Figure S3.

**Table 1:** Selected Bond Lengths and Bond Angles of [Cu(dsbtmp)_2_](PF_6_)_2_ and [Cu(dsbtmp)_2_](PF_6_)_2_

complex	bond	bond length (Å)	bond	bond angle (degree)
[Cu(dsbtmp)_2_](PF_6_)	Cu1–N1	2.022(2)	N1–Cu1–N2	81.79(9)
	Cu1–N2	2.088(2)	N4–Cu1–N3	82.02(9)
	Cu1–N3	2.074(2)	N1–Cu1–N4	139.42(9)
	Cu1–N4	2.038(2)	N2–Cu1–N3	111.42(9)
[Cu(dsbtmp)_2_](PF_6_)_2_	Cu1–N1	1.961(6)	N1–Cu1–N2	84.5(3)
	Cu1–N2	2.010(8)	N1–Cu1–N4	120.0(2)
	Cu1–N3	1.959(5)	N4–Cu1–N3	84.2(3)
	Cu1–N4	2.027(7)	N2–Cu1–N3	113.9(3)

The bite angles between nitrogen atoms on each dsbtmp ligand coordinated to the Cu(I) center are 81.78° and 82.02°, dictated by the geometry of the phenanthroline ligand backbone. However, the angles between the two nearest nitrogen atoms on neighboring dsbtmp ligands and the Cu(I) center are 111.42° and 139.42°, which is due to the steric bulk of the alkyl groups on the phenanthroline backbone, primarily the *sec*-butyl groups. For comparison, in the case of [Cu(phen)_2_]^+^_,_ the angles of the nearest neighboring N atoms and Cu(I) are 109.7° each, which is close to a tetrahedral structure.^42^ For [Cu(dsbtmp)_2_]^2+^, the angles between the two nearest nitrogen atoms on neighboring dsbtmp ligands and the Cu(II) metal center are 113.9° and 120.0°, which are surprisingly close to the Cu(I) structure. The [Cu(phen)_2_]^2+^ structure usually distorts to open up a fifth coordination site which tends to be occupied by a solvent molecule, forming a trigonal bipyramidal structure.^43−45^

The structures of the [Cu(dsbtmp)_2_]^2+/+^ complexes were determined by calculating the four-coordinate geometry index, τ_4_ as introduced by Houser et al., where a τ_4_ of 1 represents an ideal tetrahedral geometry and a τ_4_ of 0 represents an ideal square planar geometry.^37^ The index for [Cu(dsbtmp)_2_]^2+/+^ was found to be 0.7208, which indicates a pseudodisphenoidal (seesaw) geometry for both of the copper centers. Cu(I) complexes are generally tetrahedral, which undergo a Jahn–Teller distortion upon oxidation from a d^10^ to d^9^ configuration, which typically leads to flattening of the d^9^ complex. In a disphenoidal complex, however, no Jahn–Teller distortion is expected, consistent with the similar measured bond lengths and bond angles of both the Cu(I) and Cu(II) centers.

Two sets of retro cells were fabricated with electrolytes containing 0.1 M [Cu(dsbtmp)_2_](PF_6_), 0.05 M [Cu(dsbtmp)_2_](PF_6_)_2_, and 0.1 M LiPF_6_ in dry acetonitrile. TBP has been shown to induce a shift in the CB edge of TiO_2_ toward more negative potentials.^46^ Furthermore, the steric hindrance offered by the 4-*tert*-butyl group has been documented to play a significant role in reducing recombination through the shielding effect it exerts on the TiO_2_ surface.^47^ In the case of copper electrolytes in DSSCs, it has also been shown that TBP undergoes ligand substitution reactions with some Cu(II) species which results in a reduction of recombination and an improvement in device performance.^48^ Therefore, in one set of cells, 0.5 M TBP was introduced to compare the behavior to that of the control set without TBP.

**Figure 3:**
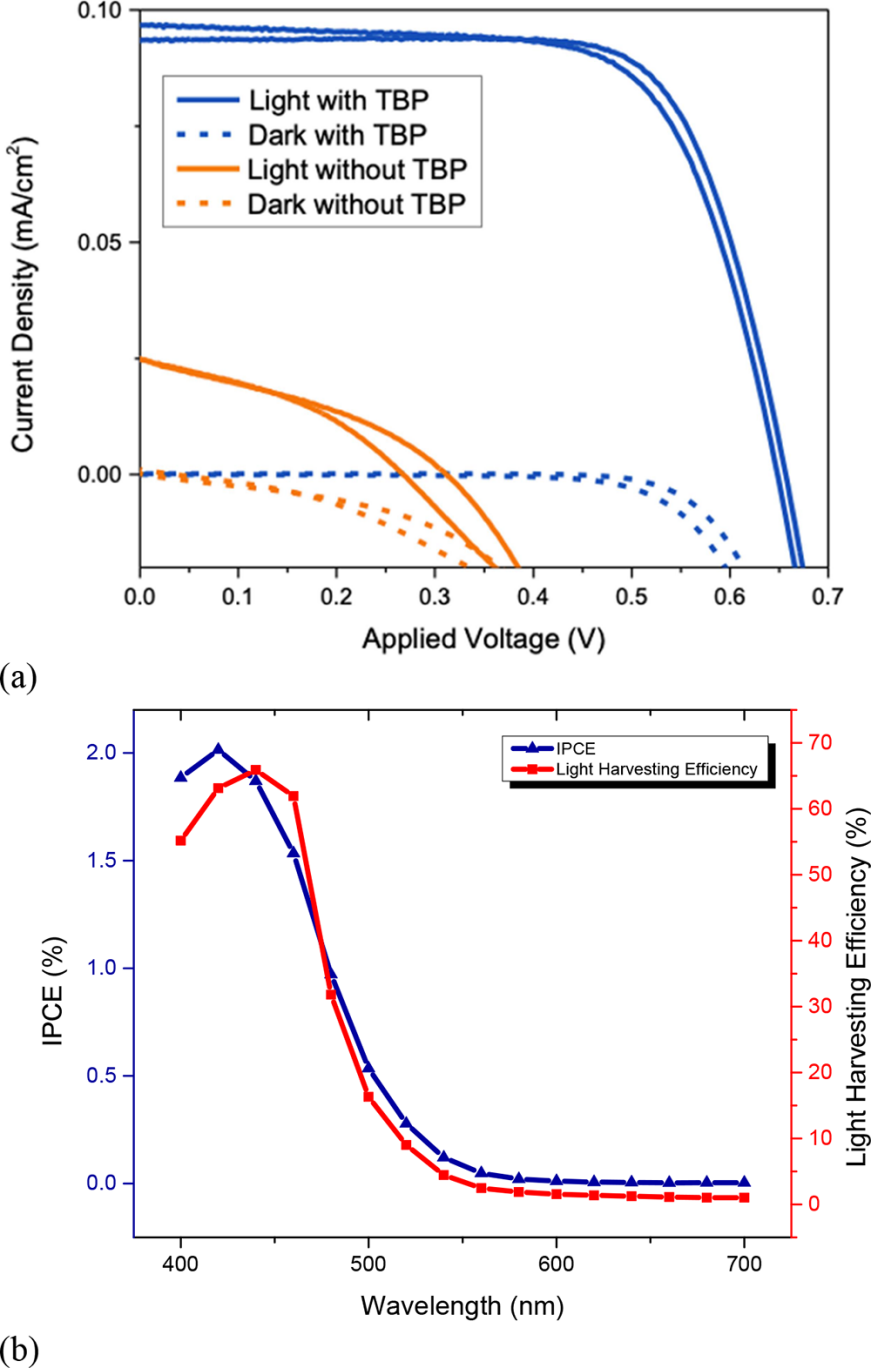
(a) Current density vs applied voltage characteristics of retro cells measured under 100 mW/cm^2^ illumination (solid lines) and under dark conditions (dotted lines) for [Cu(dsbtmp)_2_]^2+/+^ with TBP (dark blue) and without TBP (orange) in the electrolyte. (b) Incident photon-to-current conversion efficiency (blue, triangles) and light harvesting efficiency (red, squares) for [Cu(dsbtmp)_2_]^2+/+^ retro cells with TBP.

The current density vs applied voltage (*J–V*) response of representative retro cells, both with and without TBP, in the dark and under AM 1.5G (100 mW cm^–2^) light is displayed in Figure 3a. The cells with TBP showed an average open circuit voltage (*V*_OC_) of 0.65 V (±0.023 V), an average short circuit current density (*J*_SC_) of 0.097 mA/cm^2^ (±0.020 mA/cm^2^), and a PCE of 0.045%. The cells without TBP resulted in negligible values for both *V*_OC_ (0.2 V) and *J*_SC_ (0.03 mA/cm^2^) and, thus, overall efficiency. Averages and standard deviations reported here are from batches of five cells for each condition; other batches showed the same trends. Notably, the inclusion of TBP yielded a substantial improvement in device performance, emphasizing the significant role played by TBP in the cell configuration which we discuss further below.

**Figure 4:**
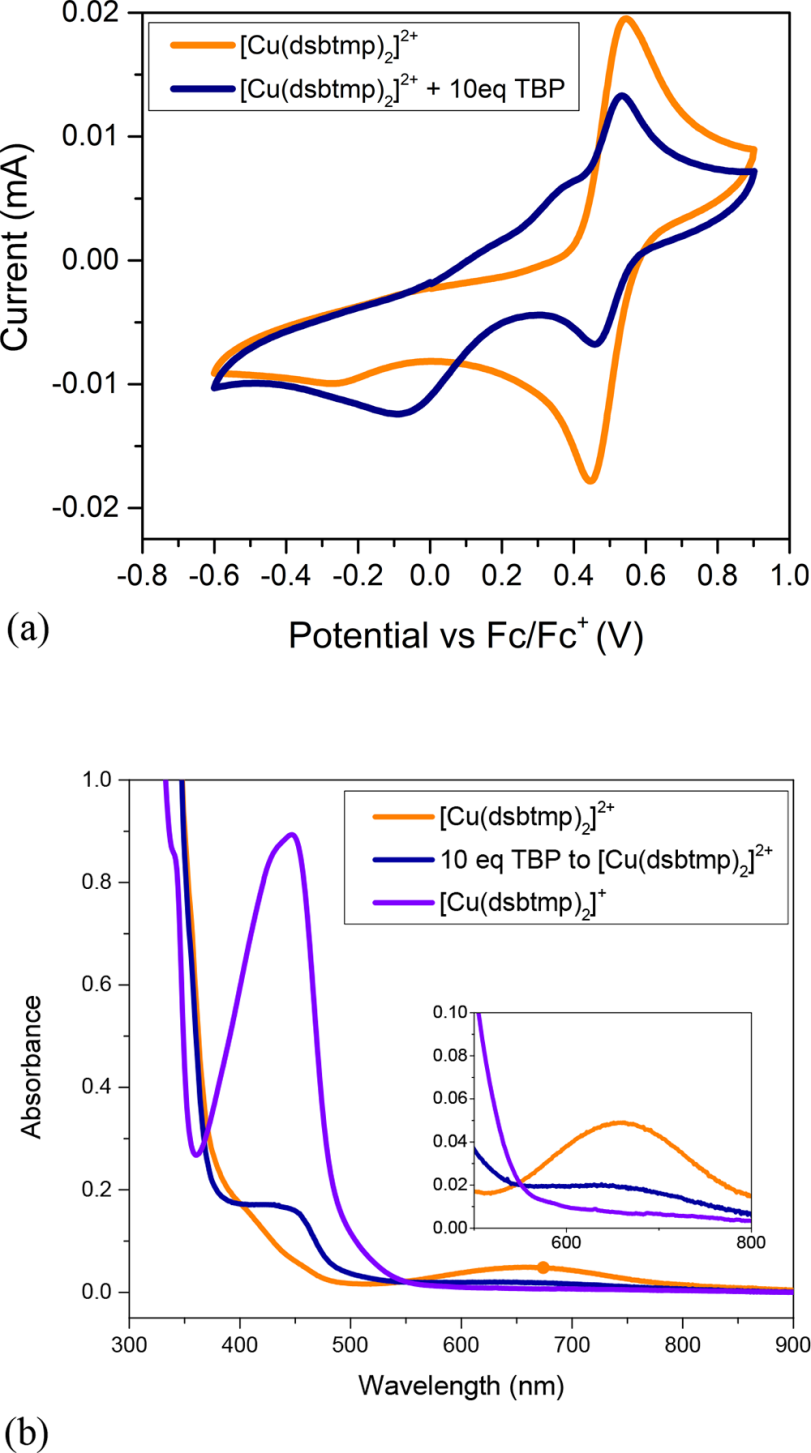
(a) Cyclic voltammogram 2 mM [Cu(dsbtmp)_2_]^2+^ in acetonitrile with and without addition of 20 mM TBP (10 equiv) at a scan rate of 0.05 V/s. (b) UV–vis spectra of 0.2 mM each of [Cu(dsbtmp)_2_]^2+^, [Cu(dsbtmp)_2_]^+^, and a mixture of 0.2 mM [Cu(dsbtmp)_2_]^2+^ and 10 eq TBP.

Incident photon to current efficiency (IPCE) measurements also were performed to assess the spectral response of DSSCs; the results are displayed in Figure 3b. The peak in the 400–450 nm region resembles the absorption spectrum of [Cu(dsbtmp)_2_]^+^ (Figure 4b), indicating that the photocurrent in the retro cells is derived from the excitation of the Cu(I) species. Control cells without the Cu(I) complex displayed negligible photocurrent/photovoltages. The current densities derived from the integration of IPCE data, Figure S2, are consistent with the *J–V* measurements, thus indicating that the IPCE captures the behavior of the *J–V* measurements. The IPCE can be described by the product of the light-harvesting efficiency, η_LH_, the charge separation efficiency, η_sep_, and the charge collection efficiency η_cc_:
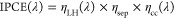
1

The light harvesting efficiency (η_LH_) is defined as the percent of incident photons absorbed by the copper chromophore within the 11 μm thick mesoporous electrode. It is determined from the absorption profile of the complex, described in detail in the Supporting Information, with results shown in Figure 3b.

The charge separation efficiency is determined by the diffusion of excited sensitizers to the TiO_2_ surface, thus the diffusion coefficient, the excited state lifetime of the sensitizers, and the injection efficiency, which are all independent of the excitation wavelength. The charge collection efficiency is controlled by recombination via back electron transfer and is a function of the electron diffusion length, *L*_n_, film thickness, *d*, and the absorption coefficient, which gives rise to a dependence on wavelength. At longer wavelengths, ca. 450–550 nm, where the absorption by Cu(I) decreases, the electrons are injected into TiO_2_ farther from the substrate, and therefore the injected electrons must diffuse longer distances to be collected. If charge collection is limited by fast recombination, then the IPCE at these wavelengths would be negligible, which is not the case. In fact, the IPCE linearly correlates to the LHE, independent of the wavelength of the incident light, indicating that the charge separation is the limiting factor for photocurrent in these retro cells and not charge collection. The open circuit photovoltage is also related to the rate of recombination; the relatively large *V*_oc_ for cells with TBP is thus also consistent with slow recombination and good charge collection.

The excited state lifetime was found to be 1.4 μs by time-resolved emission spectroscopy measurements in acetonitrile with 10 mM solution, consistent with prior reports.^39^ Control measurements showed identical emission behavior in the presence of the Cu(II) species, showing this does not quench the excited state. Measurements in the presence of TBP, however, showed a somewhat reduced excited state lifetime of 1.1 μs. Thus, the TBP does not deactivate the excited state complex by coordination, but TBP presumably has outer sphere effects similar to the prior report of a 1.2 μs lifetime in a 1:1 ACN:H_2_O solvent. The diffusion coefficient was found to be 6.2 × 10^–6^ cm^2^ s^–1^ by the analysis of cyclic voltammograms shown in Figure S11. Thus, an excited state diffusion length of 59 nm is predicted. Assuming that the TiO_2_ pore size is on the order of the nanoparticle size of ∼25 nm, all excited chromophores should collide with the TiO_2_ surface at least twice. We, therefore, suggest that the photocurrent and overall efficiency are limited by η_sep_, which we primarily attribute to a poor charge injection efficiency at contact.

The excited state potential is estimated from the following equation:
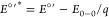
2where *E*°′ is the formal reduction potential of the ground state of the complex, *E*°′* is the formal reduction potential of the excited state, and *E*_0–0_ is the minimum energy between the ground state and the excited state. *E*°′ was found to be 0.43 V vs Fc^+^/Fc from cyclic voltammetry measurements shown in Figure 4, consistent with prior reports. *E*°′* is approximated to be the energy of emission maxima.^5^ The intersection of the absorption and the emission spectra of [Cu(dsbtmp)_2_]^+^ is shown in Figure S8. The *E*_0–0_ is ca. 300 mV more negative than the CB edge, suggesting an adequate driving force for efficient charge separation. The CB is challenging to measure accurately and is known to shift with conditions; thus, the maximum driving force above is an estimate, and additional experiments are underway to better resolve this. One possible explanation for the poor charge separation may be weak coupling between the complex and the semiconductor. As noted above, TBP has been shown to inhibit recombination by steric hindrance of acceptors to the TiO_2_ surface, which, in our case, may also inhibit injection. Elucidation of the charge injection dynamics and the effect of TBP on charge separation is the subject of ongoing investigations, but the presence of TBP has a dramatic effect on improving the photocurrent and photovoltage that cannot be explained by just shifting the band edge or steric hindrance of recombination which we attribute to improvements in charge η_cc_.

The interaction of TBP with the copper complexes may be a critical factor in enhancing the charge collection efficiency. The solution potential, *E*_s_, of the electrolyte was measured with a Pt wire without TBP and was found to be 0.420 V vs Fc^+^/Fc, consistent with expectations of the Nernst equation. TBP was titrated into the electrolyte, and the solution potential shifted negatively upon each addition of TBPs until 0.35 M was added (Table 2). Additional TBP did not further shift the solution potential. The stabilized potential was −0.131 V vs Fc^+^/Fc, representing a ∼ 500 mV change in solution potential. A negative 270 mV change in solution potential upon the addition of TBP to [Cu(dmbpy)_2_]^2+/+^ and a negative 190 mV change in solution potential for [Cu(PDTO)]^2+/+^, where PDTO is 1,8-bis(2′-pyridyl)-3,6-dithiaoctane, were also reported. These changes in solution potential were attributed to a ligand exchange reaction of the Cu(II) species to form a redox active complex, [Cu(TBP)_*x*_]^2+^, with a more negative potential.^48,49^ The large change in solution potential observed here thus also suggests a change in the composition of the redox active species in the electrolyte. Importantly, because the counter electrode is poised at the solution potential and serves as the reference in the 2-electrode sandwich device configuration, a negative shift in solution potential represents a loss in voltage. The *V*_oc_ goes up by 300 mV with TBP; however, even with the shift in *E*_s_, the Fermi level in TiO_2_ at an open circuit increases by an incredible 800 mV.

**Table 2:** Solution Potential of the Titrated Electrolytes Was Determined from Open-Circuit Potential Measurements with Both Pt and Glassy Carbon Electrodes

[TBP]/M	*E*_s_/V vs Fc^+^/Fc
0	0.420
0.05	0.401
0.10	0.359
0.15	0.299
0.20	0.194
0.25	0.163
0.30	–0.017
0.35	–0.131
0.40	–0.131
0.45	–0.131
0.50	–0.131

Titrations of the Cu(I) and Cu(II) species with TBP were, therefore, independently conducted and monitored by cyclic voltammetry and UV–vis absorption spectroscopy. TBP has no noticeable impact on the Cu(I) species on the time scales of the measurements, as seen in Figure S3. Titrations of the Cu(II) species with TBP, however, indicate a significant effect on the voltammograms and UV–vis spectra, which are shown in Figure 4a,b, respectively. In the voltammogram, the anodic and cathodic return waves diminish significantly upon the addition of TBP. In addition, a broad quasi-reversible redox wave is observed to grow in at more negative potentials. A formal potential cannot be accurately determined from the buried anodic peak. Square wave and differential pulsed voltammetry also did not reveal a clear peak associated with this species. The potential is similar to our previous report of *E*_1/2_ of 0.08 V vs Fc^+^/Fc for [Cu(TBP)_4_]^2+/+^, however.^30^ We therefore hypothesize that TBP substitutes one or both dsbtmp ligands, analogous to prior reports of the less sterically hindered [Cu(dmbpy)_2_]^2+/+^, to form [Cu(dsbtmp)(TBP)]^2+/+^ or [Cu(TBP)_4_]^2+/+^.^48^

[Cu(dsbtmp)_2_]^+^ has its characteristic MLCT peak at 450 nm, while the [Cu(dsbtmp)_2_]^2+^ absorbs at 660 nm (d-d) as seen in the UV–vis spectrum in Figure 4b. Upon titration of various equivalents of TBP to [Cu(dsbtmp)_2_]^2+^, the peak at 660 nm disappears concurrently with the growth of a new peak at 450 nm. The presence of an isosbestic point in the spectra indicates the formation of one new species from the [Cu(dsbtmp)_2_]^2+^ in the UV–vis measurement time scale. The new species cannot be attributed to [Cu(dsbtmp)_2_]^+^, as the line shape and the absorption coefficients are different. In a previous report, we showed the displacement of dmbpy ligands of [Cu(dmbpy)_2_]^2+^ by TBP to make the [Cu(TBP)_4_]^2+^ complex.^48^ In order to test this possibility, [Cu(TBP)_4_]^+/2+^ complexes were synthesized by previously reported methods.^30^ The UV–vis absorption spectra of the [Cu(TBP)_4_]^+/2+^ species are shown in Figure S5. [Cu(TBP)_4_]^2+^ has a peak at 580 nm and does not match the unknown species in Figure 4b.

**Figure 5:**
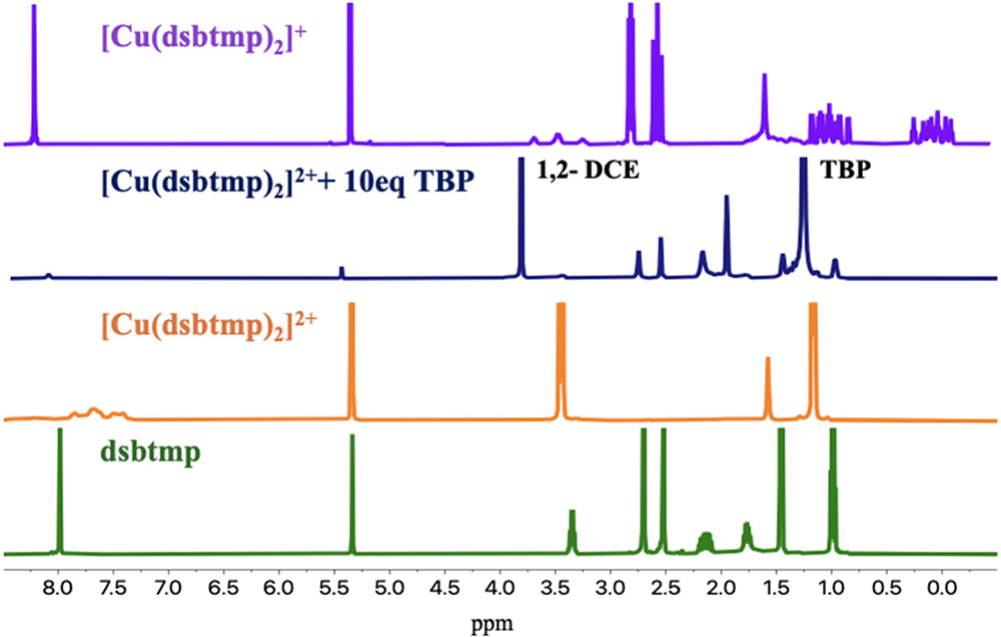
^1^H NMR (500 MHz, RT, ACN-*d*_*3*_) spectra from bottom to top: dsbtmp, [Cu(dsbtmp)_2_]^2+^, [Cu(dsbtmp)_2_]^+^ and 10 equiv of TBP, and [Cu(dsbtmp)_2_]^+^.

To investigate the unknown species formed with TBP, ^1^H NMR spectroscopy measurements were conducted on the [Cu(dsbtmp)_2_]^2+^ complex in ACN-*d*_3_, in both the presence and absence of TBP, shown in Figure 5. Due to the paramagnetic nature of [Cu(dsbtmp)_2_]^2+^, the proton signals of the dsbtmp ligands coordinated to the Cu(II) center appear significantly broadened and shifted relative to those of free dsbtmp in the solution. The full spectrum is shown in the Supporting Information. Upon introduction of TBP to the Cu(II) species, the spectra reveal the complete reappearance of all peaks assigned to free dsbtmp ligand, indicating a ligand exchange where TBP displaces dsbtmp in the coordination sphere of Cu(II), resulting in the release of free dsbtmp into solution. Quantitative ^1^H NMR studies were carried out in which 3 mM [Cu(dsbtmp)_2_]^2+^ was titrated with 10 equiv of TBP, and a known volume (1 μL) of 1,2-dichloroethane (DCE) was added to the solution. By integrating the aromatic ligand peak at 8.33 ppm and the DCE peak at 3.83 ppm, it was found that 50% of the ligand dissociates from [Cu(dsbtmp)_2_]^2+^ which implies that [Cu(dsbtmp)(TBP)_*x*_]^2+^ is the unknown product. The amount of TBP bound to the Cu(II) center was estimated by comparing the initial concentrations of Cu(II) and TBP with the amount of free TBP via comparison to the DCE peaks. The ratio of bound TBP to Cu(II) suggests the [Cu(dsbtmp)(TBP)_*x*_]^2+^ complex has x equal to ca. 4–5. This value is likely an overestimate due to the rapid ligand exchange by TBP. Attempts to isolate or independently prepare this complex to definitively determine the value of *x* and the structure of the formed complex have been unsuccessful. EPR studies were also carried out to verify the oxidation state and gain structural information on the Cu center in the [Cu(dsbtmp)(TBP)_*x*_]^2+^ species. The EPR spectrum, shown in Figure S10, has *gx*, *gy*, and *gz* values of 2.2806, 2.1305, and 2.0243 and an *R*-value of 0.7075 which is indicative of a Cu(II) center, thus ruling out a Cu(I) species being formed and is consistent with the proposed product [Cu(dsbtmp)(TBP)_*x*_]^2+^.

## Conclusions

In conclusion, we have shown the viability of a retro cell utilizing [Cu(dsbtmp)_2_]^2+/+^ to function as both the chromophore and the redox shuttle in a mesoporous TiO_2_ electrode. The long excited-state lifetime of [Cu(dsbtmp)_2_]^+^ is a critical parameter to allow diffusion of the excited chromophore to the TiO_2_ surface for charge separation. The charge separation efficiency is found to be poor, however, which is attributed to the charge injection step and the possible deleterious effect of TBP. The addition of TBP was found to be crucial, however, in allowing good charge collection and large photovoltages to be achieved. In addition to TBP controlling the band edge position and blocking recombination, we found that TBP displaces one dsbtmp ligand on the Cu(II) complex formed following charge separation, which results in a purported poor electron acceptor complex that also minimizes recombination. The competing roles of TBP found in this first report suggest the possibility of selectively controlling the ligand exchange to allow good charge collection without deleterious charge injection through variation of exogenous base and ligand framework, which is the key focus of our current research.

## Experimental Section

All reagents were sourced from commercial suppliers and used as received without further purification. Tetramethyl-1,10-phenanthroline, toluene, *sec*-butyl-lithium, dichloromethane, manganese dioxide, magnesium sulfate, *n*-hexane, tetrakis(acetonitrile)copper(I) hexafluorophosphate, diethyl ether, nitrosonium hexafluorophosphate, titanium(IV) chloride solution, ethanol, hydrochloric acid (HCl), and hexachloroplatinic(IV) acid (H_2_PtCl_6_) were obtained from Sigma-Aldrich. Celite was obtained from Jade Scientific.

2,9-Di(*sec*-butyl)-3,4,7,8-tetramethyl-1,10-phenanthroline, dsbtmp, and bis(2,9-di(*sec*-butyl)-3,4,7,8-tetramethyl-1,10-phenanthroline)copper(I) hexafluorophosphate, [Cu(dsbtmp)_2_](PF_6_) were made following protocols established in the literature.^39^ Detailed procedure, characterization, and spectra are provided in the Supporting Information. Bis(2,9-di(*sec*-butyl)-3,4,7,8-tetramethyl-1,10-phenanthroline)copper(II) bis(hexafluorophosphate) ([Cu(dsbtmp)_2_](PF_6_)_2_) was prepared by adding a solution of nitrosonium hexafluorophosphate, NOPF_6_ (0.15 g, 0.24 mmol) in 2 mL dichloromethane to a solution of [Cu(dsbtmp)_2_](PF_6_) (0.2 g, 0.24 mmol) in 2 mL of dichloromethane, and the reaction mixture was stirred for 30 min. The product was precipitated using diethyl ether and then filtered. The yield of this reaction was 78% (0.19 g). Elemental analysis: found (calcd) for C_48_H_64_CuF_12_N_4_P_2_: C, 54.94 (54.88); H, 6.28 (6.14); N, 5.39 (5.33).

Retro cell electrodes were made following previously reported procedures,^44^ without the sensitization step. Briefly, the working electrode consists of TEC 8 fluorine-doped tin oxide (FTO) glass substrates purchased from Pilkington, coated with an 11 μm thick layer of 30 nm TiO_2_ nanoparticles by doctor blading a commercial paste (DSL 30NRD) purchased from Greatcell Solar Materials Pty Ltd., followed by annealing. The platinized counter electrode was made by depositing a 5 mM H_2_PtCl_6_ solution in isopropanol on FTO glass substrates followed by heating at 380 °C for 20 min. The electrodes are sandwiched together using 25 μm Surlyn films purchased from Solaronix. Electrolytes consisted of 0.1 M Cu(I), 0.05 M Cu(II), and 0.1 M lithium hexafluorophosphate (LiPF_6_) in dry acetonitrile. Some electrolytes, as noted, also contained 0.5 M TBP. Cells were measured approximately 1 h after fabrication. Six solar cells were measured for each electrolyte condition, and the average and standard deviation were reported.

Solar cell measurements were made using a Horiba Jobin Yvon 450 W xenon arc light with an AM 1.5 solar filter. IPCE measurements used a Horiba Jobin Yvon MicroHR monochromator. Electrochemical measurements were performed using a μAutolabIII potentiostat with a three-electrode system: glassy carbon as the working electrode, platinum mesh as a counter electrode, and Ag/AgNO_3_ as the reference electrode in an inert atmosphere glovebox. The electrolyte solution potentials were determined by the open circuit potential between a platinum wire and the Ag/AgNO_3_ electrode immersed in the electrolyte. Absorption spectra were acquired by using a PerkinElmer Lambda 35 UV–vis spectrometer and 1 cm path length quartz cuvettes at 480 nm min^–1^. Steady-state emission spectra were obtained using the Horiba Jobin Yvon Fluorolog Spectrofluorometer with excitation at 450 nm. Time-resolved emission measurements were carried out in the Edinburgh Instruments LP980 transient absorption spectrometer.

## Supporting Information Available

Details of synthetic methods and characterization of ligands and complexes reported herein, additional crystal structures, light harvesting efficiency calculations, and spectroscopic and electrochemical measurement results (PDF)
