# Supplementary material for: Single Component Dye-Sensitized Solar Cells Enabled by Copper Chemistry: Introduction of the Retro Cell
Source: Energy Fuels. 2025 Mar 7;39(11):5604–11. doi: 10.1021/acs.energyfuels.4c06413 (PMC11931484; doi:10.1021/acs.energyfuels.4c06413)
Supplement: Supplementary file 1 — ef4c06413_si_001.pdf [file ef4c06413_si_001.pdf]

Supporting Information

Single Component Dye Sensitized Solar Cells Enabled by Copper Chemistry; Introduction of the Retro Cell

Samhita Kaushik, Michael A. Adesanya and Thomas W. Hamann*

Department of Chemistry, Michigan State University

East Lansing, Michigan 48824-1322 United States

orcid.org/0000-0001-6917-7494 (hamann@chemistry.msu.edu)

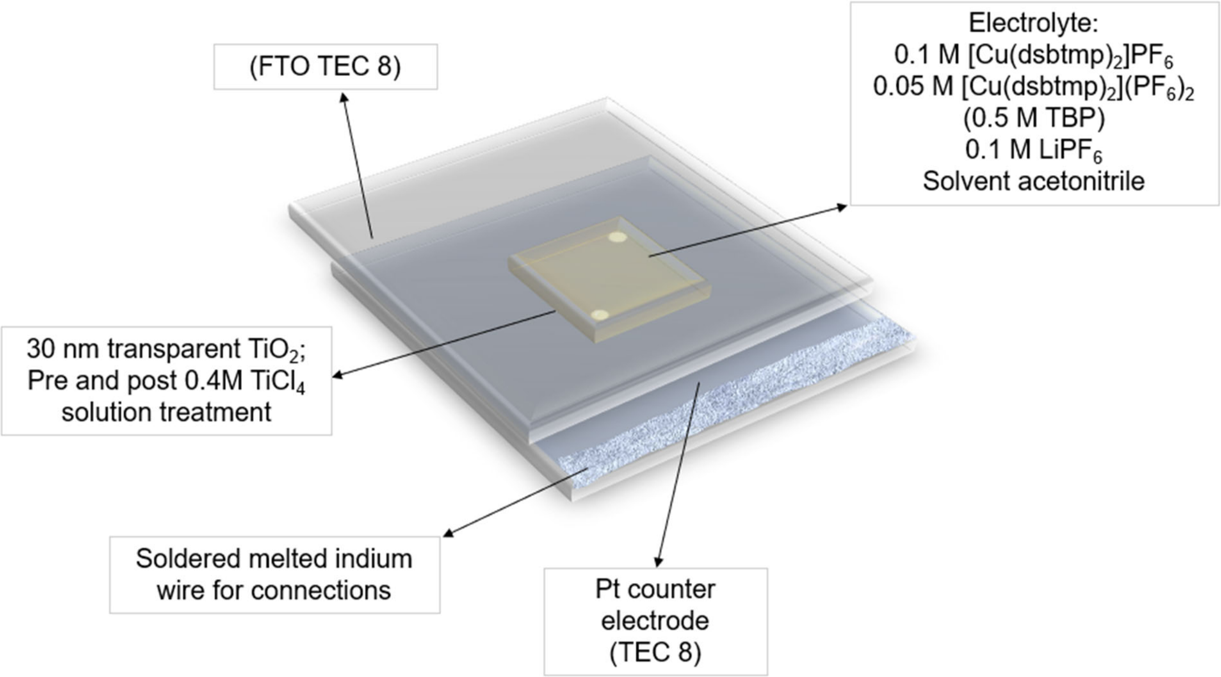

Figure S1: Representation of the components of a Retro Cell

Methods

Materials

All reagents were sourced from commercial suppliers and used as received without further purification. Tetramethyl-1,10-phenanthroline, toluene, sec-butyl-lithium, dichloromethane, manganese dioxide, magnesium sulfate, *n*-hexane, tetrakis(acetonitrile)copper(I) hexafluorophosphate, diethyl ether, nitrosonium hexafluorophosphate, titanium(IV) chloride solution, ethanol, hydrochloric acid (HCl) and hexachloroplatinic(IV) acid (H_2PtCl_6) were obtained from Sigma Aldrich. Celite was obtained from Jade Scientific.

2,9-di(sec-butyl)-3,4,7,8-tetramethyl-1,10-phenanthroline, dsbtmp, and bis(2,9-di(sec-butyl)-3,4,7,8-tetramethyl-1,10-phenanthroline)copper(I) hexafluorophosphate, $[Cu(dsbtmp)_2](PF_6)$ were made following protocols established in the literature.[8] Specific modifications to the synthetic procedures have been detailed in the following section.

Synthesis

2,9-Di(sec-butyl)-3,4,7,8-tetramethyl-1,10-phenanthroline (dsbtmp)

Tetramethyl-1,10-phenanthroline (8.46 mmol, 2g) was suspended in 30 mL of anhydrous toluene and then was reacted with sec-butyl-lithium (30 ml of 1.45 M) under nitrogen atmosphere at 0°C and stirred overnight at room temperature. The reaction was quenched with water (20.0 ml) and the organic layer was separated. The aqueous layer was extracted three times with dichloromethane (20.0 ml), and the combined organic layers were treated with excess manganese dioxide (50.0 g). This was stirred overnight then gravity filtered through celite. The filtrate was dried with magnesium sulfate, filtered and the solvent was removed by rotary evaporation. The product was recrystallized with n-hexane. The yield of this reaction is 67% (1.67 g, 4.79 mmol). ^{1}H NMR (RT, 500 MHz, $CDCl_3$) δ 7.91 (s, 2H), 3.29 (q, *J* = 6.7, 2H), 2.66 (s, 6H), 2.49 (s, 6H), 2.24 – 2.06 (m, 2H), 1.77 (m, 2H), 1.48 (dd, *J* = 6.7, 4.4 Hz, 6H), 0.99 (m, 6H).

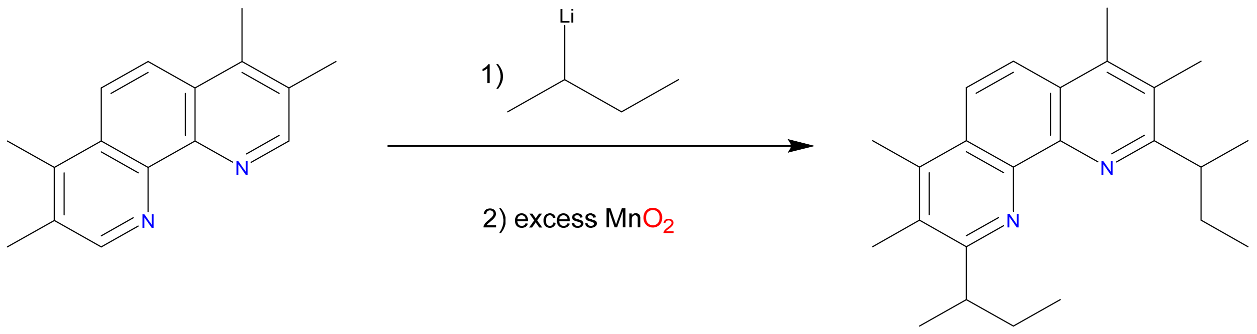

***Scheme S1**: Synthesis of 2,9-di(sec-butyl)-3,4,7,8-tetramethyl-1,10-phenanthroline*

Bis(2,9-di(sec-butyl)-3,4,7,8-tetramethyl-1,10-phenanthroline)copper(I) hexafluorophosphate ($[Cu(dsbtmp)_2](PF_6)$)

Tetrakis(acetonitrile)copper(I) hexafluorophosphate (0.260 g, 0.68 mmol) and dsbtmp (0.5 g, 1.43 mmol) were added to 10mL DCM. The solution was stirred overnight, and the product was precipitated with diethyl ether. The product was then obtained by decantation. The yield of this reaction is 80.4% (0.49 g, 0.54 mmol). ^{1}H NMR (RT, 500 MHz, $CDCl_3$) δ 8.21 (s, 2H), 3.42 – 3.72 (m, 2H), 2.71 (m, 6H), 2.54 (t, J = 19.1 Hz, 6H), 1.28 (d, J = 13.1 Hz, 4H), 1.12 – 0.77 (m, 6H), 0.24 – -0.17 (m, 6H). Elemental analysis: found (calculated) for $C_{48}H_{64}CuF_6N_4P$: C, 63.99 (63.66); H, 7.26 (7.12); N, 6.16 (6.19).

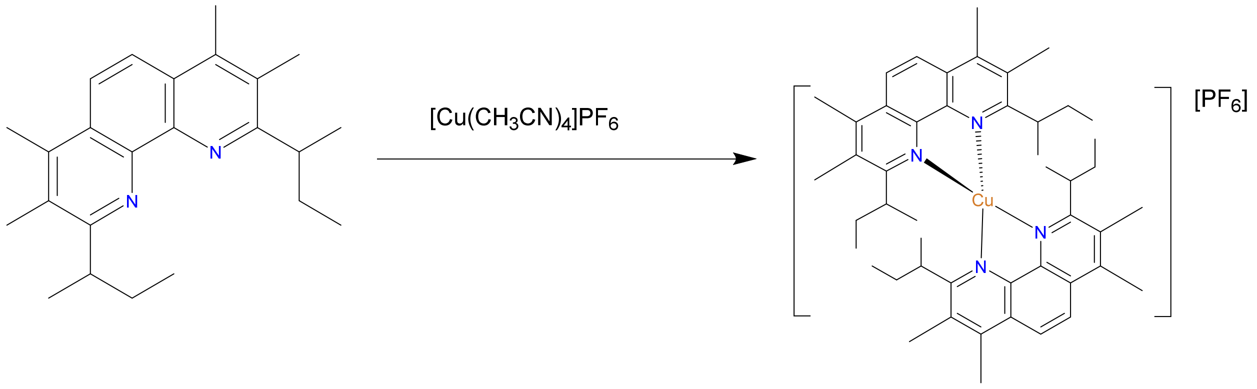

***Scheme S2**: Synthesis of bis(2,9-di(sec-butyl)-3,4,7,8-tetramethyl-1,10-phenanthroline)copper(I) hexafluorophosphate*

Bis(2,9-di(sec-butyl)-3,4,7,8-tetramethyl-1,10-phenanthroline)copper(II) bis(hexafluorophosphate) ([Cu(dsbtmp)$_2$](PF$_6$)$_2$)

A solution of [Cu(dsbtmp)$_2$](PF$_6$) (0.2 g, 0.24 mmol) of 2mL of dichloromethane was prepared. Another solution of nitrosonium hexafluorophosphate, $NOPF_6$ (0.15 g, 0.24 mmol) in 2mL dichloromethane was prepared. Both the solutions were mixed, and the reaction was stirred for 30 minutes. The product was precipitated using diethyl ether and then filtered. The yield of this reaction was 78% (0.19 g, 0.18 mmol). Elemental analysis: found (calculated) for $C_{48}H_{64}CuF_{12}N_4P_2$: C, 54.94 (54.88); H, 6.28 (6.14); N, 5.39 (5.33).

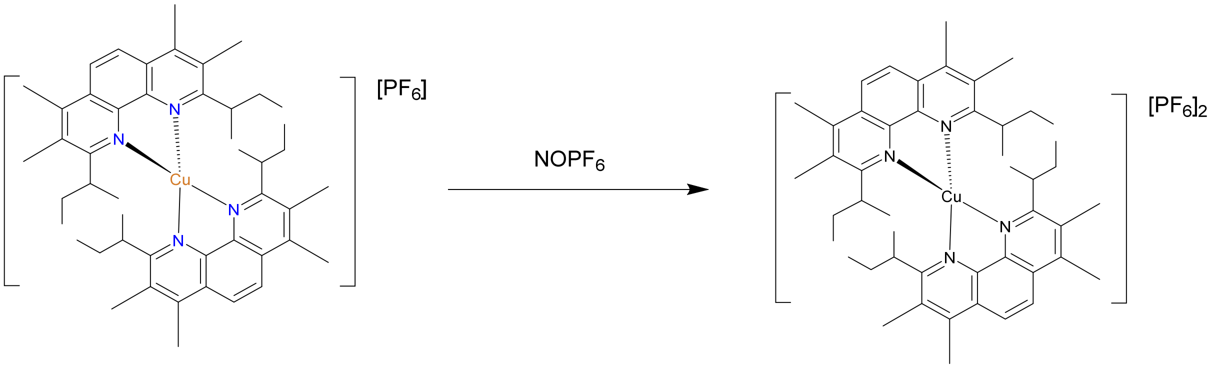

***Scheme S3**: Synthesis of bis(2,9-di(sec-butyl)-3,4,7,8-tetramethyl-1,10-phenanthroline)copper(II) bis(hexafluorophosphate)*

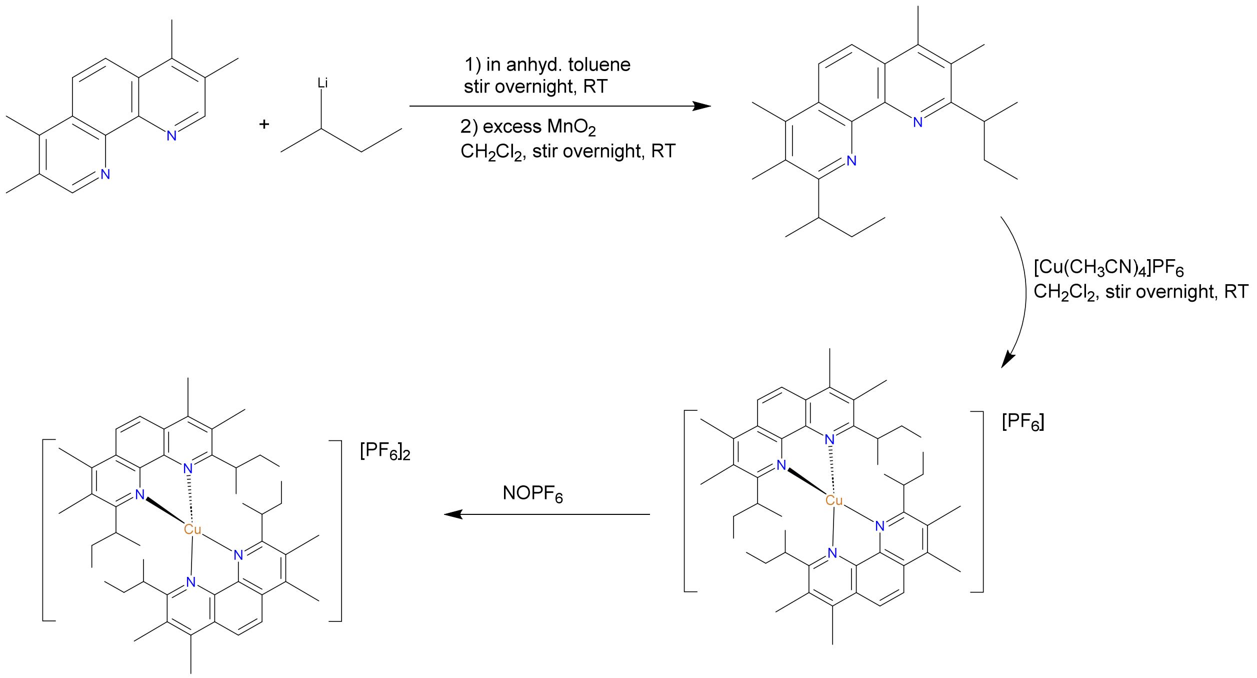

Device Fabrication

A retro cell consists of a fluorine doped tin oxide (FTO) glass substrate layered with TiO_2 that functions as the photoanode. The thickness of the TiO_2 layer was 11 μm as determined by profilometry. Another FTO glass substrate layered with Pt catalyst acts as the cathode. These glass substrates are sandwiched together, and the electrolyte is injected through pre-drilled holes on the cathode. The holes are sealed with a cover slip using Surlyn sheets as the sealant. FTO-coated glass (8 Ω sq^{-1} (TEC 8)) was purchased from Pilkington North America. TiO_2 paste was purchased from Greatcell Solar Materials Pty Ltd. Surlyn with a dimension of 25 μm was procured from Solaronix. The TEC 8 FTO substrates were sectioned into pieces measuring 1.5 cm by 2 cm. These sections underwent a 15-minute sonication process in soapy DI water, followed by manual scrubbing using Kimwipes. Subsequently, a 10-minute sonication in DI water, along with rinsing in acetone and a 10-minute sonication in isopropanol, was carried out. After air-drying, the sections were immersed in an aqueous 40 mM $TiCl_4$ solution at 70 °C for 30 minutes. Following this, the sections were promptly rinsed with 18 MΩ water and subjected to annealing, involving heating from room temperature to 500 °C, followed by a 30-minute hold at 500 °C.

A templated area of 0.36 cm^2 was layered with a commercial 30 nm TiO_2 nanoparticle paste (DSL 30NRD) by doctor blading. The resultant transparent films were allowed to sit undisturbed for a period of 10 minutes before being placed within a 100 °C oven for 15 minutes. The oven's temperature was then incrementally increased, reaching 325 °C for 5 minutes, followed by 375 °C for 5 minutes, 450 °C for 5 minutes, and finally, 500 °C for 15 minutes.

Platinum counter electrodes were prepared by the following steps: first, two holes were drilled into a sheet of TEC 8 FTO. The FTO was then sonicated in a soap solution, followed by a 5-minute sonication in DI water. It was then rinsed with a 0.1M HCl solution in ethanol (~1 mL concentrated HCl in 100 ml) and subjected to a 10-minute sonication in an acetone bath. Following this, any residual organic contaminants were removed by heating the material in air at approximately 400ºC for about 15 minutes. The next step was coating it with around 0.5 drop/cm^2 of a 5mM H_2PtCl_6 solution in isopropanol. The material was then quickly tilted to spread the solution and allowed to dry without exposure to a breeze for a duration of 5 minutes. Lastly, the material was subjected to a heat treatment at 380ºC for 20 minutes, after which it was removed from the heat source and allowed to cool while covered in ambient conditions. The assembly process involved sandwiching the working and counter electrodes using 25 µm surlyn films. This was accomplished by placing them onto a hotplate at 140 °C and applying pressure while the Surylyn melted. Contact was established with the TiO_2 electrode by gently scratching its edge with sandpaper and then applying melted indium. Similarly, melted indium was applied to the counter electrode. The indium was left to dry for 15 minutes. Then, the cells were filled with electrolyte via one of two predrilled holes and then sealed. This sealing process was executed by employing 25 µm surlyn, backed by a glass coverslip, and applying heat to achieve an airtight seal.

Two sets of electrolytic conditions were measured. For the first set, the electrolyte consisted of 0.1 M Cu(I), 0.05 M Cu(II) and 0.1 M lithium hexafluorophosphate ($LiPF_6$) in dry acetonitrile. For the second set, the electrolyte consisted of 0.1 M Cu(I), 0.05 M Cu(II),0.1 M $LiPF_6$ and 0.5 M 4-*tert*-butyl pyridine (TBP) in dry acetonitrile. Cells were measured approximately 1 hour after fabrication. Six solar cells were measured for each electrolyte condition.

Instrumentation

An AM 1.5 solar filter was employed to replicate sunlight conditions at 100 mW cm^{-2}, and the light intensity was calibrated using an accredited reference cell system (Oriel Reference Solar Cell & Meter). In order to prevent direct excitation of the TiO_2 during light measurements, a 400 nm long-pass filter was incorporated. A black mask, with an open area measuring 0.12 cm^2, was placed on the cell which acts as the active region. The devices' solution potential was determined by immersing a platinum wire and the $Ag/AgNO_3$ electrode into the electrolyte, while subsequently measuring the potential between these electrodes. For monochromatic light used in IPCE (incident photon-to-current efficiency) measurements, a monochromator (Horiba Jobin Yvon MicroHR) coupled with a 450 W xenon arc light source was used. The photon flux of the light directed onto the samples was measured via a laser power meter (Nova II Ophir). IPCE measurements were conducted in 20 nm intervals across the range of 400 to 600 nm, focusing on the short circuit current.

Electrochemistry

All the electrochemical measurements were performed using 0.1M lithium hexafluorophosphate in acetonitrile. They were carried out in an inert atmosphere in the glovebox. The cyclic voltammetry measurements were performed using a μAutolabIII potentiostat with a three-electrode system- glassy carbon as the working electrode, platinum mesh as a counter electrode and $Ag/AgNO_3$ as the reference electrode. The cyclic voltammetry measurements were carried out using 2mM solution of each complex.

Optical Spectroscopy

Absorption spectra for the complexes were acquired using a PerkinElmer Lambda 35 UV-vis spectrometer and 1 cm path length quartz cuvettes at 480 nm min^{-1}. Solution concentration of 0.2mM was used for each complex. Steady state emission spectra were obtained using Horiba Jobin Yvon Fluorolog Spectrofluorometer. The measurements were performed at room temperature. The sample was excited at 450 nm.

Time resolved emission measurements for the complexes were carried out in Edinburgh Instruments LP980 transient Absorption Spectrometer. The excitation wavelength was 650 nm.

The measurements were carried out using 10mM solution of each complex using acetonitrile as the solvent. The sample was excited at 450 nm and emission was detected at 650 nm.

Single Crystal X-Ray Crystallography

Single crystals were grown in dichloromethane with slow diethyl ether diffusion over the course of three days. A suitable crystal with dimensions 0.12 × 0.12 × 0.05 mm^3 was selected and mounted on a nylon loop with paratone oil on a XtaLAB Synergy, Dualflex, HyPix diffractometer. The crystal was kept at a steady T = 100.00(10) K during data collection. The structure was solved with the ShelXT (Sheldrick, 2015) solution program using dual methods and by using Olex2 1.3 (Dolomanov et al., 2009) as the graphical interface. The model was refined with ShelXL 2018/3 (Sheldrick, 2015) using full matrix least squares minimization on F^2.

Light harvesting efficiency (LHE)

LHE was calculated using the following equation:

A= -logT, where A is the absorbance and T is the transmittance of the incident light.

LHE= 1-R-T, where R is the reflectance of the substrate. The reflectance of the electrodes were previously found to be ca 20% for wavelength range of interest here. [1]

Transmittance is the ratio of transmitted light through the solar cell to the total incident light on the solar cell. About 80% of the incident light goes through the TiO_2/electrolyte layer, thus the equation becomes:

$$LHE= 1-R-T = 0.8 - T = 0.8 - 0.8\,(10^{-\epsilon*C*l*P})$$

where P is the porosity of the TiO_2 film (50%), ϵ is the molar absorptivity of the electrolyte, C is the concentration of the electrolytic solution, l is the path length of light through TiO_2 film measured by profilometry (11 μm).

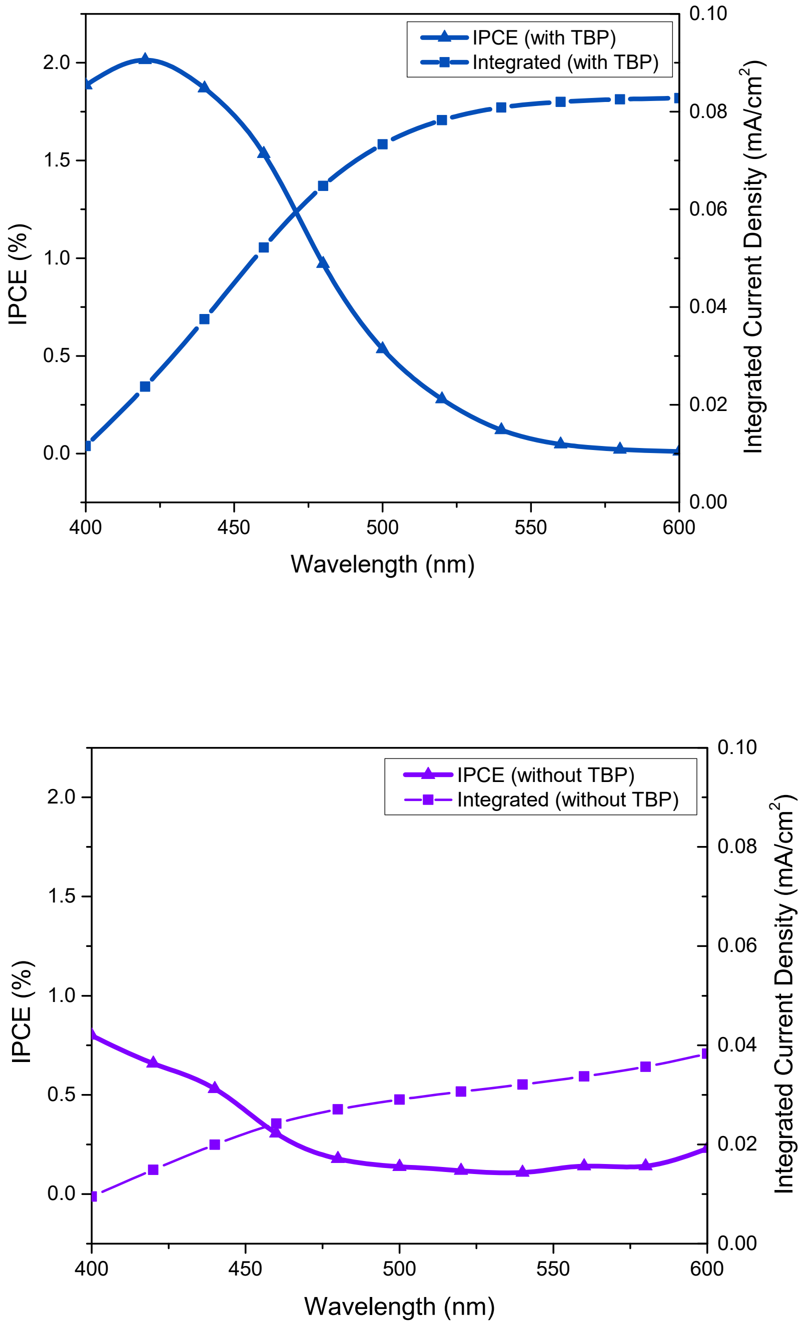

Figure S2: Incident photon-to-current conversion efficiency (triangles) and integrated current density (solid line) for $[Cu(dsbtmp)_2]^{+/2+}$ retro cells with TBP (top) and without TBP (bottom)

Single Crystal X-Ray Crystallography

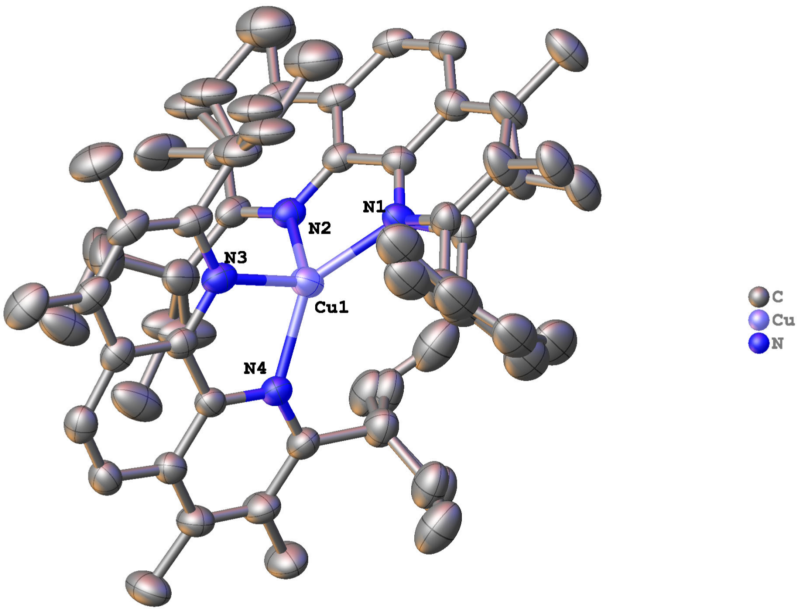

(a)

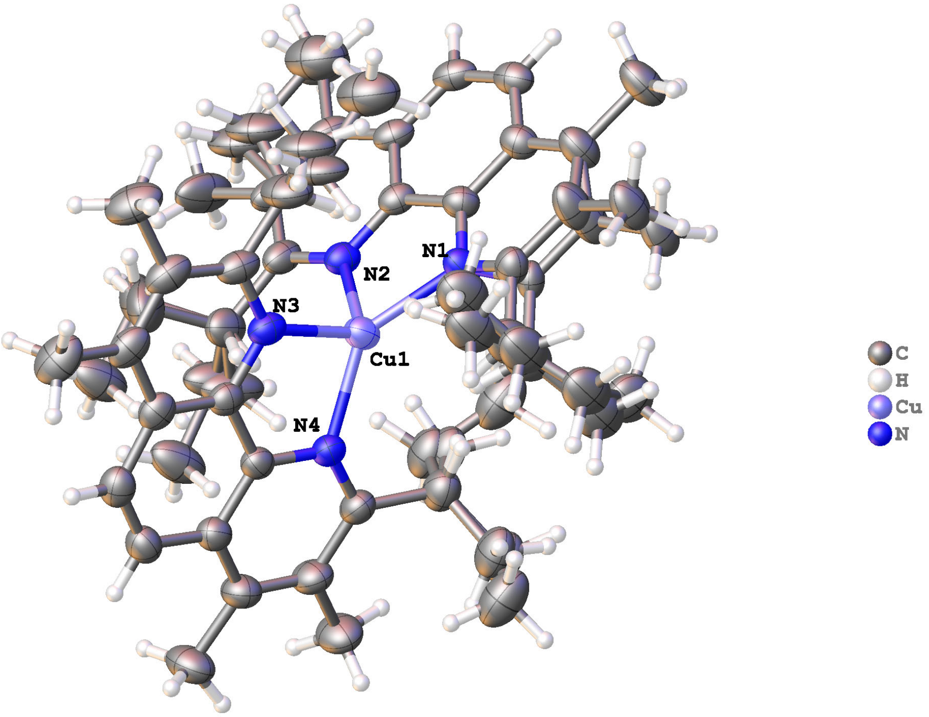

(b)

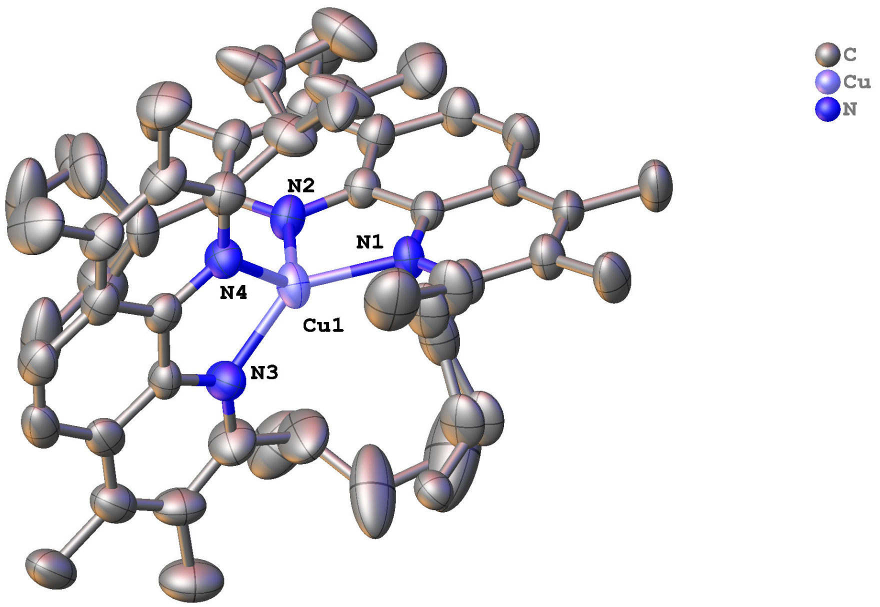

(c)

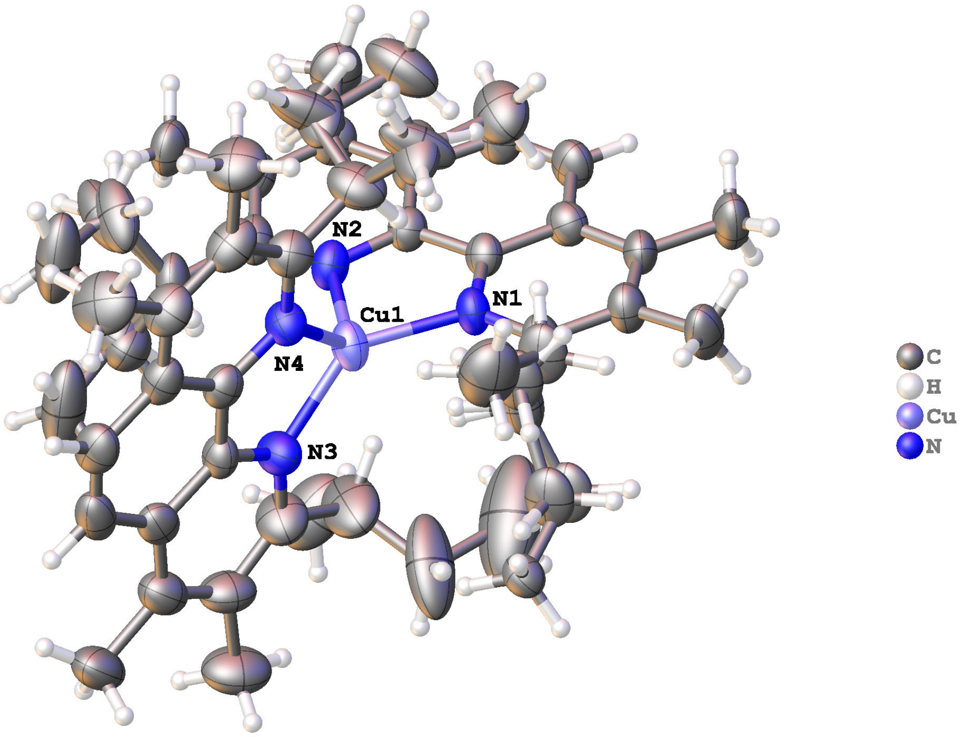

(d)

Figure S3: Additional single crystal X-Ray structures with thermal ellipsoids drawn at 50% probability of (a,c) [Cu(dsbtmp)$_2$](PF$_6$) and (b,d) [Cu(dsbtmp)$_2$](PF$_6$)$_2$ to show structural disorder in sec-butyl groups. Hydrogen atoms or omitted in a &c, and included in b & d. Counter ions are omitted for clarity.

Optical Spectroscopy

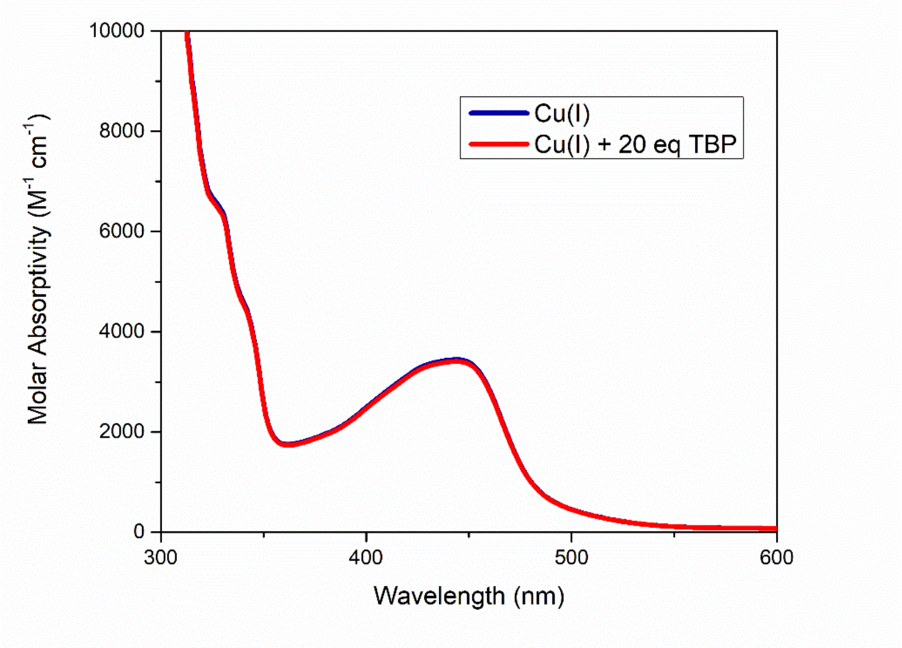

Figure S4: UV-Vis spectra showing titration of 20 equiv. TBP to 0.25 mM $[Cu(dsbtmp)_2]^+$ in acetonitrile

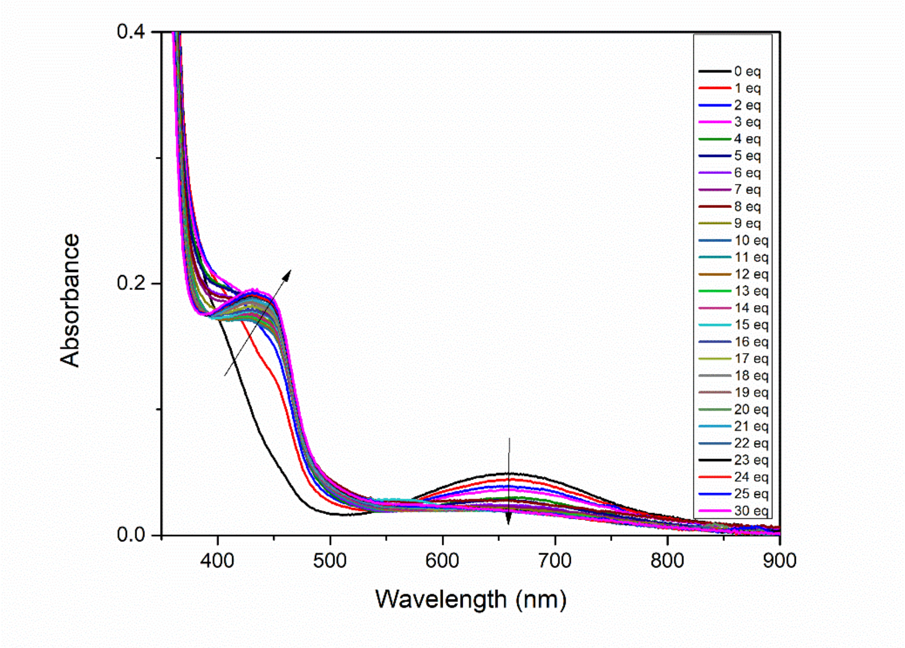

Figure S5: UV-Vis spectra showing titration of TBP (arrow indicates increase in equivalents by 1 from 0 equiv. to 30 equiv.) to 0.25 mM $[Cu(dsbtmp)_2]^{2+}$ in acetonitrile

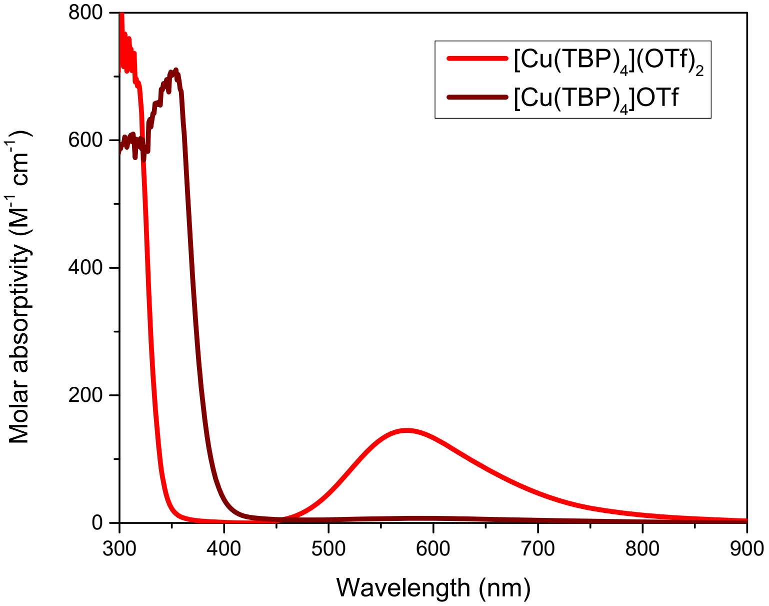

Figure S6: UV-Vis spectra of $[Cu(TBP)_4]^{2+/+}$in acetonitrile

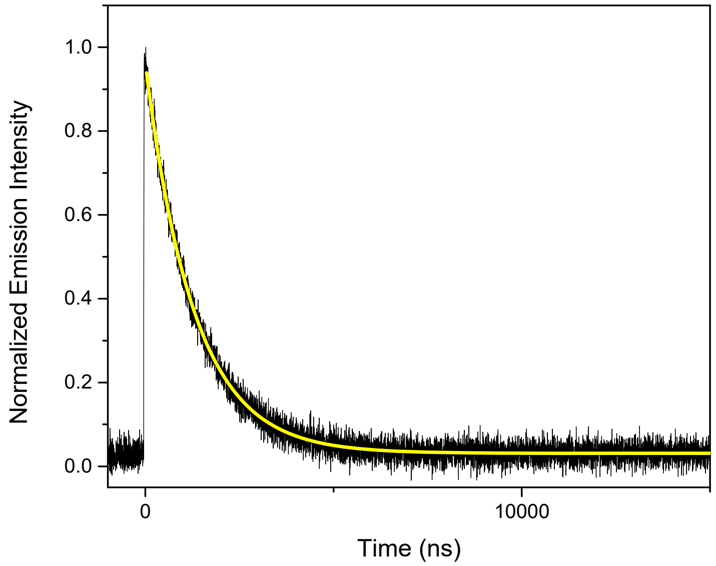

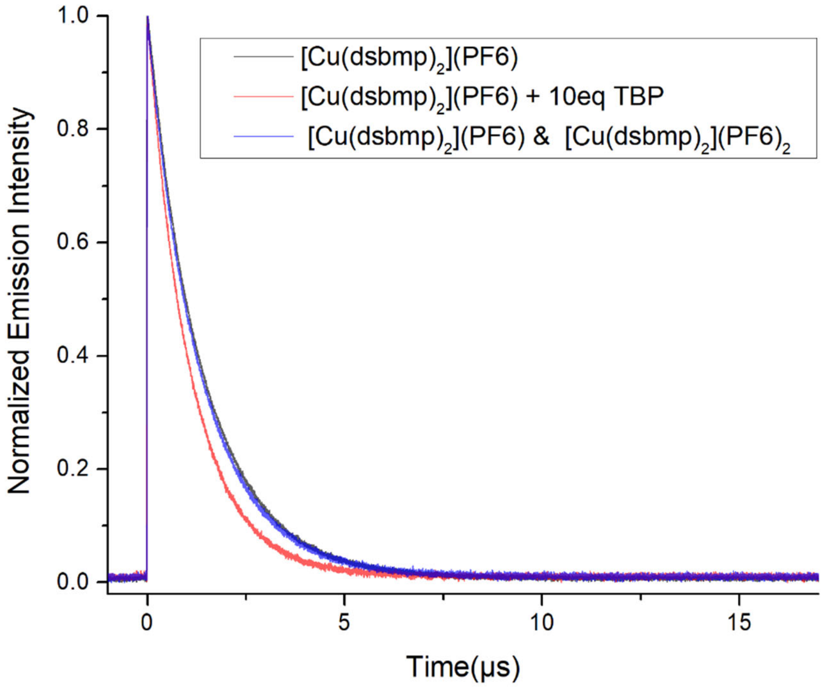

Figure S7: *Top:* Time-resolved emission decay data of 2mM $[Cu(dsbtmp)_2](PF_6)$ in acetonitrile. The sample was excited at 450 nm and emission was detected at 650 nm. *Bottom:* Normalized time-resolved emission decays of $[Cu(dsbtmp)_2](PF_6]$, $[Cu(dsbtmp)_2](PF_6]$ + 10eq TBP and $[Cu(dsbtmp)_2](PF_6)$ in presence of $[Cu(dsbtmp)_2](PF_6)_2$ in CD_3CN

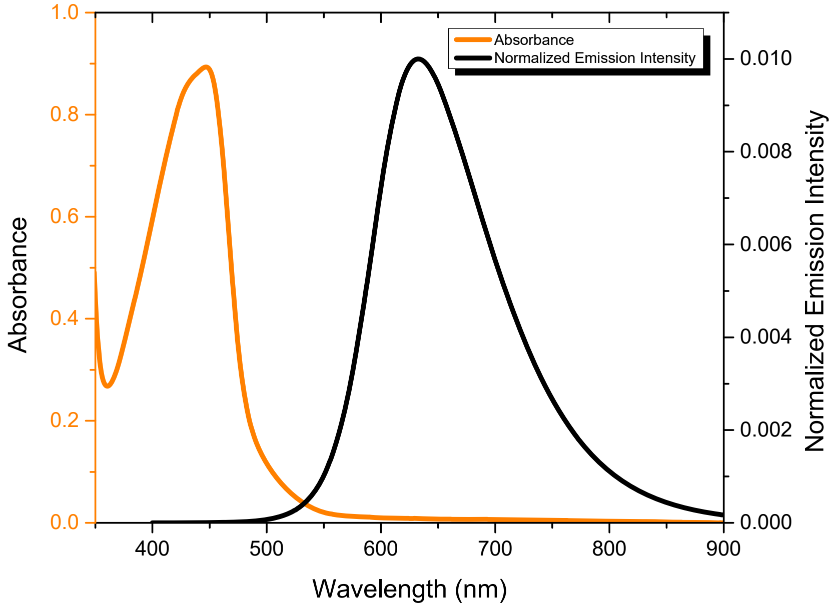

Figure S8: Overlap of steady-state emission spectrum of 2mM [Cu(dsbtmp)$_2$](PF$_6$) in acetonitrile (black) and absorption spectrum of 2mM [Cu(dsbtmp)$_2$](PF$_6$) in acetonitrile (orange).

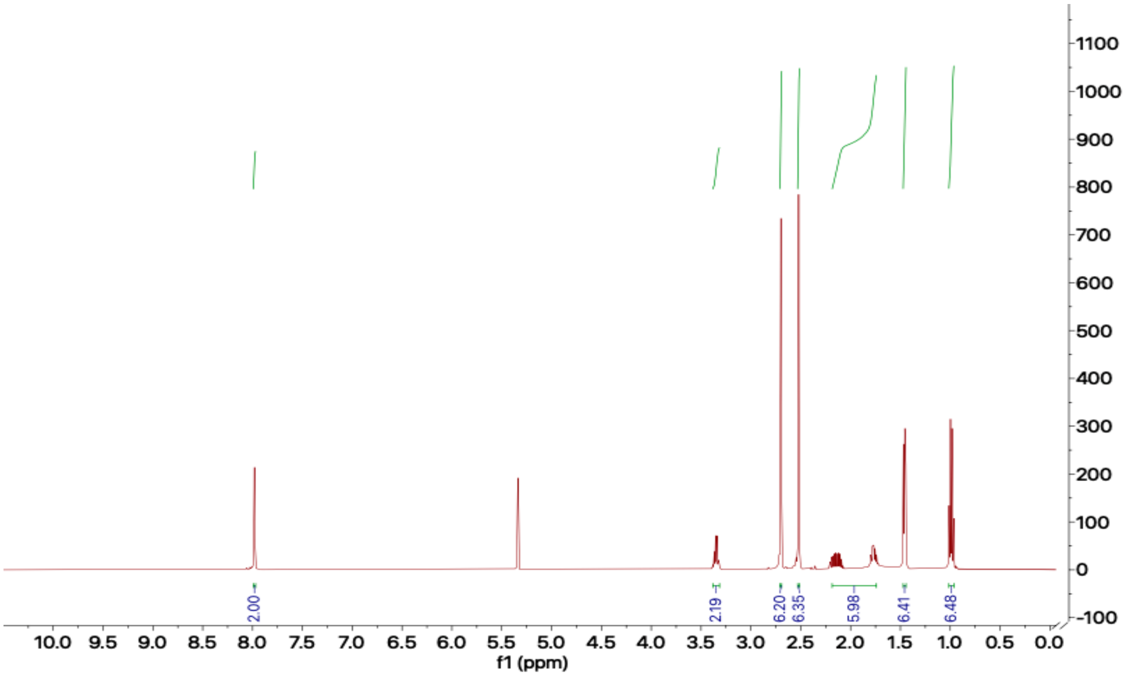

Figure S9: ^{1}H-NMR (500 MHz, RT) spectrum of free dsbtmp ligand in CD_2Cl_2

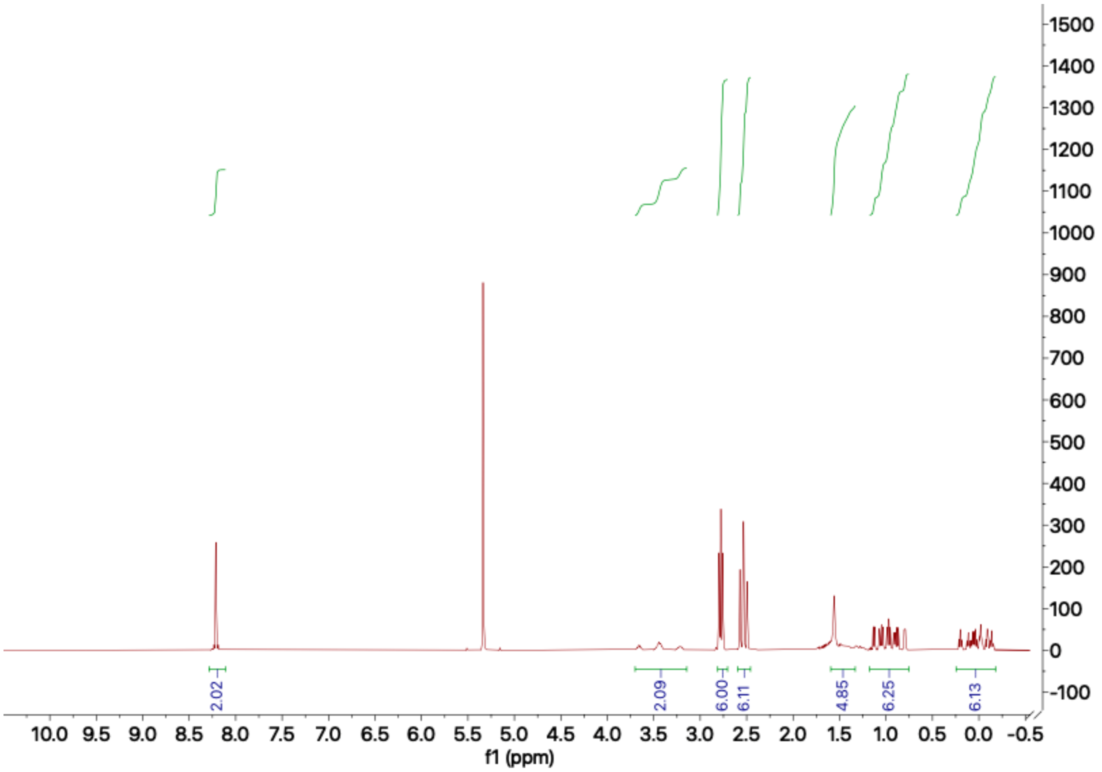

Figure S10: ^{1}H-NMR (500 MHz, RT) spectrum of $[Cu(dsbtmp)_2](PF_6)$ in CD_2Cl_2

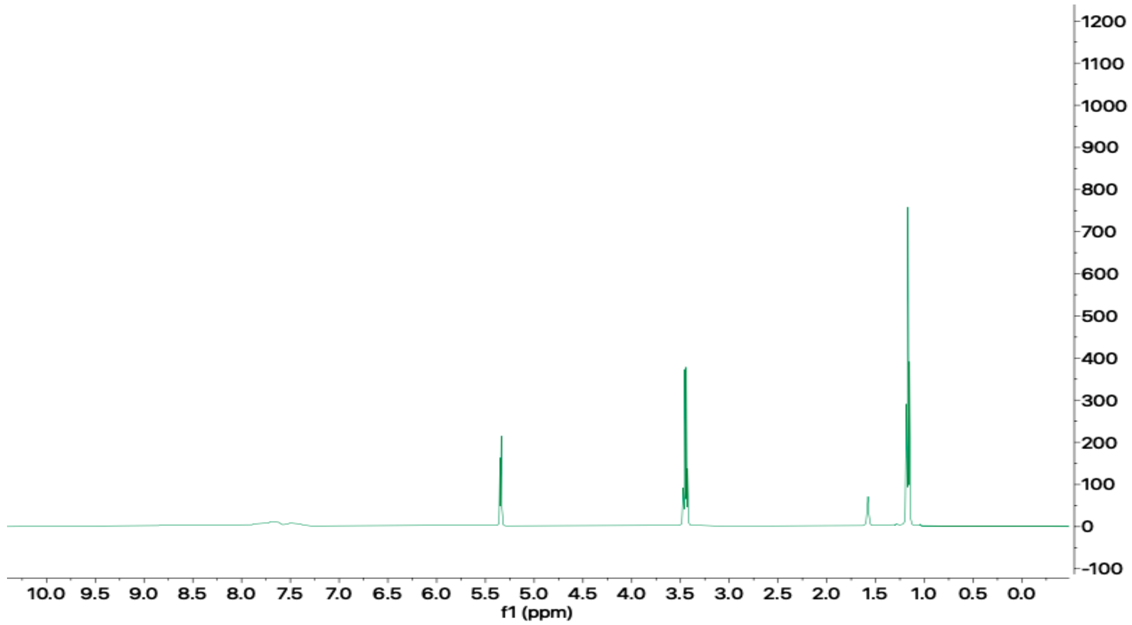

Figure S11: ^{1}H-NMR (500 MHz, RT) spectrum of $[Cu(dsbtmp)_2](PF_6)_2$ in CD_2Cl_2

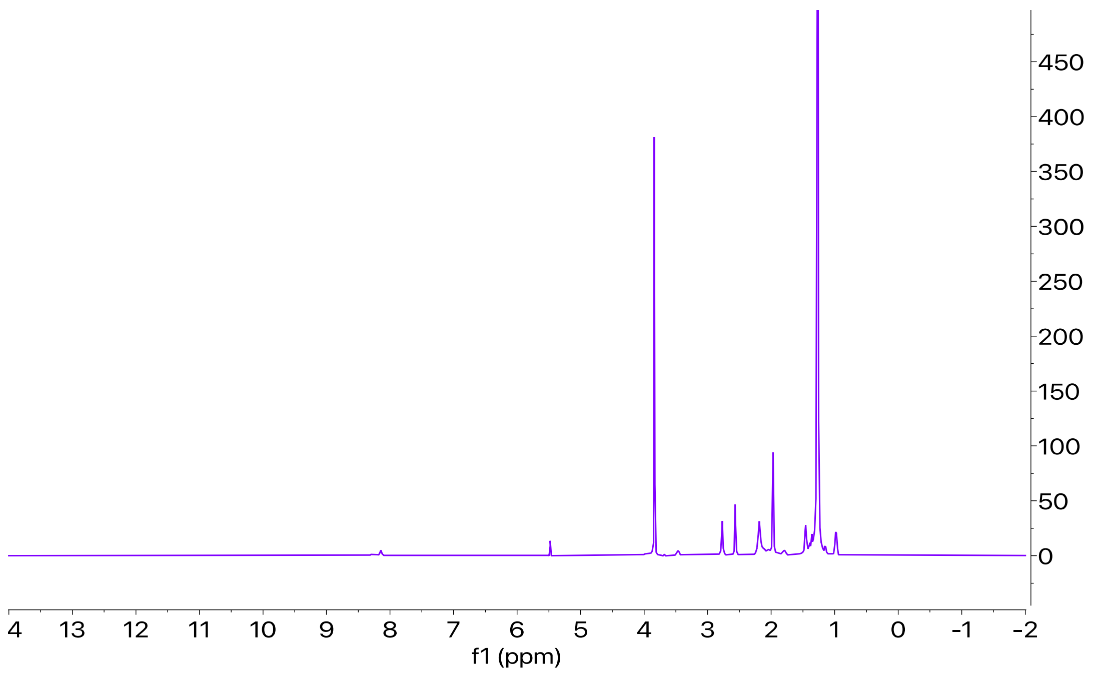

Figure S12: ^{1}H-NMR (500 MHz, RT) of a mixture of 1,2-dicloroethane, 10 equiv. TBP and 3mM $[Cu(dsbtmp)_2]^{2+}$in deuterated acetonitrile

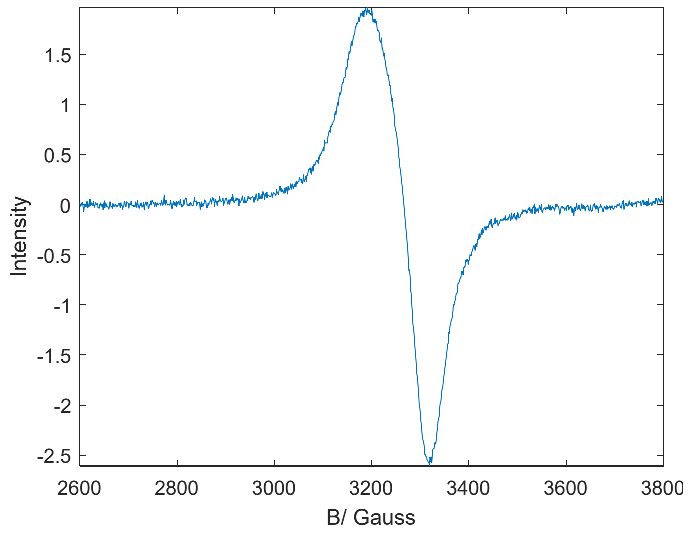

Figure S13: EPR of a mixture of $[Cu(dsbtmp)_2]^{2+}$ and 20 equiv. TBP in acetonitrile.

Cyclic voltammetry

Cyclic voltammograms of $[Cu(dsbtmp)_2](PF_6)$ were measured at different scan rates, as shown in Figure S11 (a). The peak current vs square root of scan rate was plotted, shown in Figure S11 (b). The potentials are reported relative to $Fc^{+/0}$.

$$i_p = 0.446nFAC^0 \left(\frac{nFvD}{RT}\right)^{1/2} \quad (1)$$

where n is the number of electrons transferred in the redox reaction, A is the surface area of the electrode, C^0 is the concentration of the bulk solution and D is the diffusion coefficient of analyte. Equation 1 and the slope of the plot in Figure S12 (b) was used to calculate the diffusion coefficient, which was found to be 6.2 x 10^{-5} cm^2 s^{-1}.

The diffusion length or the distance of diffusion of a species is given by the following equation[3]:

$$L = (D.\tau)^{1/2} \quad (2)$$

where L is the diffusion length; D is the diffusion coefficient of the species and τ is the lifetime in seconds. For excited $[Cu(dsbtmp)_2](PF_6)$, the diffusion length is found to be 60 nm. The lifetime of the complex was measured to be 1.5 μs which aligned with that reported in literature.[2]

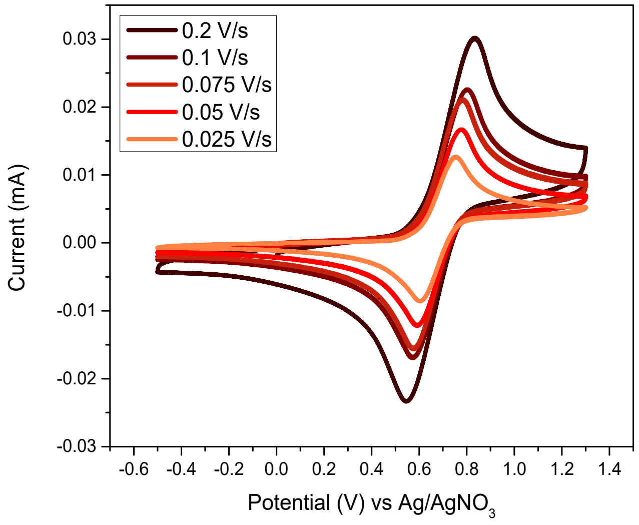

(a)

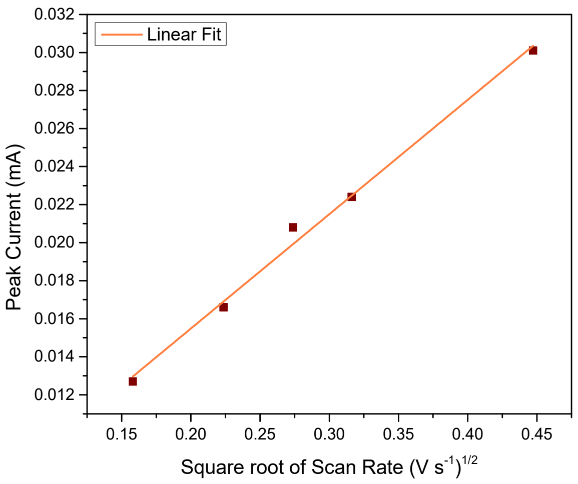

(b)

Figure S14 (a): Cyclic voltammogram of $[Cu(dsbtmp)_2]^{2+}$in acetonitrile with different scan rates shown in legend (b) Scan rate dependence (peak current vs square root of scan rate) of $[Cu(dsbtmp)_2]^{2+}$in acetonitrile

References

(1) Xie, Y.; Baillargeon, J.; Hamann, T. W. Kinetics of Regeneration and Recombination Reactions in Dye-Sensitized Solar Cells Employing Cobalt Redox Shuttles. *Journal of Physical Chemistry C* **2015**, *119* (50), 28155–28166. https://doi.org/10.1021/ACS.JPCC.5B08244.

(2) McCusker, C. E.; Castellano, F. N. Design of a Long-Lifetime, Earth-Abundant, Aqueous Compatible Cu(I) Photosensitizer Using Cooperative Steric Effects. *Inorg Chem* **2013**, *52* (14), 8114–8120. https://doi.org/10.1021/ic401213p.

(3) Bard, A. J.; Faulkner, L. R. Allen J. Bard and Larry R. Faulkner, Electrochemical Methods: Fundamentals and Applications , New York: Wiley, 2001, 2nd Ed. *Russian Journal of Electrochemistry* **2002**, *38* (12), 1364–1365. https://doi.org/10.1023/A:1021637209564.
